# Portable Breath-Based Volatile Organic Compound Monitoring for the Detection of COVID-19 During the Circulation of the SARS-CoV-2 Delta Variant and the Transition to the SARS-CoV-2 Omicron Variant

**DOI:** 10.1001/jamanetworkopen.2023.0982

**Published:** 2023-02-28

**Authors:** Ruchi Sharma, Wenzhe Zang, Ali Tabartehfarahani, Andres Lam, Xiaheng Huang, Anjali Devi Sivakumar, Chandrakalavathi Thota, Shuo Yang, Robert P. Dickson, Michael W. Sjoding, Erin Bisco, Carmen Colmenero Mahmood, Kristen Machado Diaz, Nicholas Sautter, Sardar Ansari, Kevin R. Ward, Xudong Fan

**Affiliations:** 1 Department of Biomedical Engineering, University of Michigan, Ann Arbor; 2 Max Harry Weil Institute for Critical Care Research and Innovation, University of Michigan, Ann Arbor; 3 Department of Electrical Engineering and Computer Science, University of Michigan, Ann Arbor; 4 Department of Emergency Medicine, University of Michigan, Ann Arbor; 5 Department of Internal Medicine, Division of Pulmonary Critical Care Medicine, University of Michigan, Ann Arbor

## Abstract

**Importance:**

Breath analysis has been explored as a noninvasive means to detect COVID-19. However, the impact of emerging variants of SARS-CoV-2, such as Omicron, on the exhaled breath profile and diagnostic accuracy of breath analysis is unknown.

**Objective:**

To evaluate the diagnostic accuracies of breath analysis on detecting patients with COVID-19 when the SARS-CoV-2 Delta and Omicron variants were most prevalent.

**Design, Setting, and Participants:**

This diagnostic study included a cohort of patients who had positive and negative test results for COVID-19 using reverse transcriptase polymerase chain reaction between April 2021 and May 2022, which covers the period when the Delta variant was overtaken by Omicron as the major variant. Patients were enrolled through intensive care units and the emergency department at the University of Michigan Health System. Patient breath was analyzed with portable gas chromatography.

**Main Outcomes and Measures:**

Different sets of VOC biomarkers were identified that distinguished between COVID-19 (SARS-CoV-2 Delta and Omicron variants) and non–COVID-19 illness.

**Results:**

Overall, 205 breath samples from 167 adult patients were analyzed. A total of 77 patients (mean [SD] age, 58.5 [16.1] years; 41 [53.2%] male patients; 13 [16.9%] Black and 59 [76.6%] White patients) had COVID-19, and 91 patients (mean [SD] age, 54.3 [17.1] years; 43 [47.3%] male patients; 11 [12.1%] Black and 76 [83.5%] White patients) had non–COVID-19 illness. Several patients were analyzed over multiple days. Among 94 positive samples, 41 samples were from patients in 2021 infected with the Delta or other variants, and 53 samples were from patients in 2022 infected with the Omicron variant, based on the State of Michigan and US Centers for Disease Control and Prevention surveillance data. Four VOC biomarkers were found to distinguish between COVID-19 (Delta and other 2021 variants) and non–COVID-19 illness with an accuracy of 94.7%. However, accuracy dropped substantially to 82.1% when these biomarkers were applied to the Omicron variant. Four new VOC biomarkers were found to distinguish the Omicron variant and non–COVID-19 illness (accuracy, 90.9%). Breath analysis distinguished Omicron from the earlier variants with an accuracy of 91.5% and COVID-19 (all SARS-CoV-2 variants) vs non–COVID-19 illness with 90.2% accuracy.

**Conclusions and Relevance:**

The findings of this diagnostic study suggest that breath analysis has promise for COVID-19 detection. However, similar to rapid antigen testing, the emergence of new variants poses diagnostic challenges. The results of this study warrant additional evaluation on how to overcome these challenges to use breath analysis to improve the diagnosis and care of patients.

## Key Points

**Question:**

Can volatile organic compounds (VOCs) in exhaled breath provide diagnostic information on COVID-19 disease and SARS-CoV-2 variants?

**Findings:**

In this diagnostic study with 167 participants, 4 VOC biomarkers were found to distinguish between the Delta variant of SARS-CoV-2 (and other variants occurring in 2021) from non–COVID-19 illness. The emergence of the Omicron variant in 2022 substantially affected the VOC profiles, requiring a different set of VOCs to distinguish between the Omicron variant and non–COVID-19 illness.

**Meaning:**

These findings demonstrate the ability of breath analysis to distinguish between COVID-19 and non–COVID-19 illness, but they also reveal the significant variations in the breath profile among patients with COVID-19 as new SARS-CoV-2 variants emerge.

## Teaser

This diagnostic study evaluates the accuracies of breath analysis on detecting patients with COVID-19 in periods when the Delta and Omicron variants were most prevalent.

## Introduction

The COVID-19 pandemic continues to present diagnostic challenges due to emerging variants of SARS-CoV-2. The 2 major platforms for COVID-19 diagnosis are reverse transcriptase–polymerase chain reaction (RT-PCR) and rapid antigen testing (RAT). While RT-PCR is the criterion standard, RAT was developed to address the need for faster turnaround and scaling of testing to the public for both asymptomatic and symptomatic individuals. However, the transition of the pandemic from the SARS-CoV-2 Delta variant to the Omicron variant has reduced the accuracy of RATs, thus presenting substantial challenges in their ability to maintain accuracy, which is critical to decision-making.^1,2,3,4^

Since the pandemic’s beginning, alternative methods of detecting COVID-19 have been explored, including gas chromatography–ion mobility spectrometry (GC-IMS), Fourier-transform infrared spectroscopy, GC–mass spectrometry (GC-MS), and others.^5,6,7,8,9,10,11,12,13,14,15,16,17,18,19,20^ The basis for these approaches is that breath contains hundreds of volatile organic compounds (VOCs), many of which are produced in response to inflammation and infection.^19,21,22,23,24,25,26,27,28,29,30,31,32,33,34,35,36^ Several technologies have been demonstrated to have accuracies comparable with RT-PCR testing and RATs, and 1 has recently been approved for use under the US Food and Drug Administration’s Emergency Use Authorization.^10,17,18,37^

However, nearly all results reported in these studies^5,6,7,8,9,10,11,12,13,14,15,16,17,18,19,20^ were prior to 2022, ie, when the dominant SARS-CoV-2 strain was the Delta variant. Only one recent study found differences in breath profiles between the Delta and Omicron waves.^38^ In this study, we report the use of portable GC developed as a point-of-care diagnostic modality for COVID-19 and its performance during the Delta surge and its transition to Omicron, including future challenges in using breath analysis in the current pandemic and future respiratory illness epidemics.

## Methods

### Clinical Study Protocol and Participants

This project was developed under the National Institutes of Health’s Screening for COVID-19 by Electronic-Nose Technology program in response to the public health emergency issued by the Department of Health and Human Services for COVID-19 as part of the Rapid Acceleration of Diagnostics–Radical initiative.^39^ The study was approved by the University of Michigan’s Institutional Review Board. Recruited patients included both those who received mechanical ventilation and those who could breathe spontaneously. Informed written consent was obtained from patients or their legally authorized representative. The study followed the Standards for Reporting Diagnostic Accuracy (STARD) reporting guideline.

Patients were enrolled between April 26, 2021, and May 31, 2022, via intensive care units and the emergency department at the University of Michigan Health System. Convenience sampling was used to enroll patients undergoing RT-PCR testing who were symptomatic for COVID-19 or were required to undergo RT-PCR COVID-19 screening for infection control purposes. Samples for RT-PCR tests were collected using nasopharyngeal swabbing for the patients who could breathe spontaneously and using either nasopharyngeal swabbing or tracheal aspirate for those who were receiving mechanical ventilation. The hospital uses several RT-PCR platforms, including Molecular Simplexa (DiaSorin), ID NOW (Abbott), Alinity (Abbott), and FilmArray Respiratory Panel 2.1 (Biofire), to detect SARS-CoV-2. No attempt was made to control for which RT-PCR test was used.

Participant race and ethnicity are reported as a requirement of the National Institute of Health Rapid Acceleration of Diagnostics–Radical initiative. It was determined from the patient’s registration data located in the health system’s electronic medical record.

### Breath Collection and Analysis

Breath collection and analysis was conducted within 18 hours of the corresponding RT-PCR test. For patients receiving mechanical ventilation, 1 to 2 L of breath was collected into a 5-L Tedlar bag through a T-connector attached to the ventilator’s expiratory port (eFigure 1A in Supplement 1). Patients breathing spontaneously were asked to orally exhale 1 to 2 L of breath into a 5-L Tedlar bag via a mouthpiece and a medical-grade HEPA filter (eFigure 1B in Supplement 1). Breath analysis took place in the patient’s room immediately after collection. The Tedlar bag was connected to the GC device (eFigure 1C in Supplement 1). Approximately 350 mL of breath was pulled from the bag into the GC, which was controlled remotely via a laptop. Details are described in eAppendix 1 in Supplement 1. On completion of breath analysis, consumables (T-connector, bag, mouthpiece, and HEPA filter) were disposed of. All other parts were disinfected using Oxy-bleach wipes.

### Statistical Analysis

The data analysis pipeline was developed in house and was implemented using Matlab version R2021a (MathWorks). Approximately 90 peaks were detected in each breath chromatogram. Collectively, there were a total of 131 different peaks in the 205 chromatograms. Each peak represents 1 VOC or 1 set of co-eluted VOCs. Linear discriminant analysis (LDA) and principal component analysis (PCA), which have been tested and validated in our earlier breath analysis studies on other diseases,^21,22,23,40^ were used for data set dimensionality reduction, biomarker selection, and statistical analyses. Details of the biomarker discovery algorithm were previously reported.^21^ The primary analytic outcome was binary. In the Results section, we report 4 scenarios: (1) COVID-19 (2021), which refers to patients recruited before December 14, 2021, and were assumed to be infected by Delta or earlier variants,^41,42^ vs non–COVID-19 illness, which refers to patients recruited throughout the study; (2) COVID-19 (2022), which refers to patients recruited from January 11, 2022, to the end of the study (May 31, 2022) and were therefore assumed to be infected by the Omicron variant based on the sharp transition from Delta to Omicron, as determined by surveillance testing by the state of Michigan (eFigure 4C in Supplement 1) as well as the Centers for Disease Control and Prevention (CDC)^41,42,43^ vs non–COVID-19 illness; (3) COVID-19 (2021) vs COVID-19 (2022); and (4) COVID-19 (2021 and 2022) vs non–COVID-19 illness, as well as the corresponding specificity, sensitivity, positive predictive value (PPV), negative predictive value (NPV), and accuracy (defined as the [true positive + true negative] / total number of samples). A gap of nearly 1 month between our last recruitment in December 2021 and the first recruitment in January 2022 worked as a buffer zone in the separation of the Delta and Omicron variants. At this time, the State of Michigan reported that Omicron was responsible for nearly 100% of COVID-19 infections (eFigure 4C in Supplement 1).

A subset of collected breath samples were analyzed simultaneously by our portable GC and an Agilent MS (eFigure 3A in Supplement 1). Details appear in eAppendix 2 in Supplement 1.

## Results

**Figure 1.**
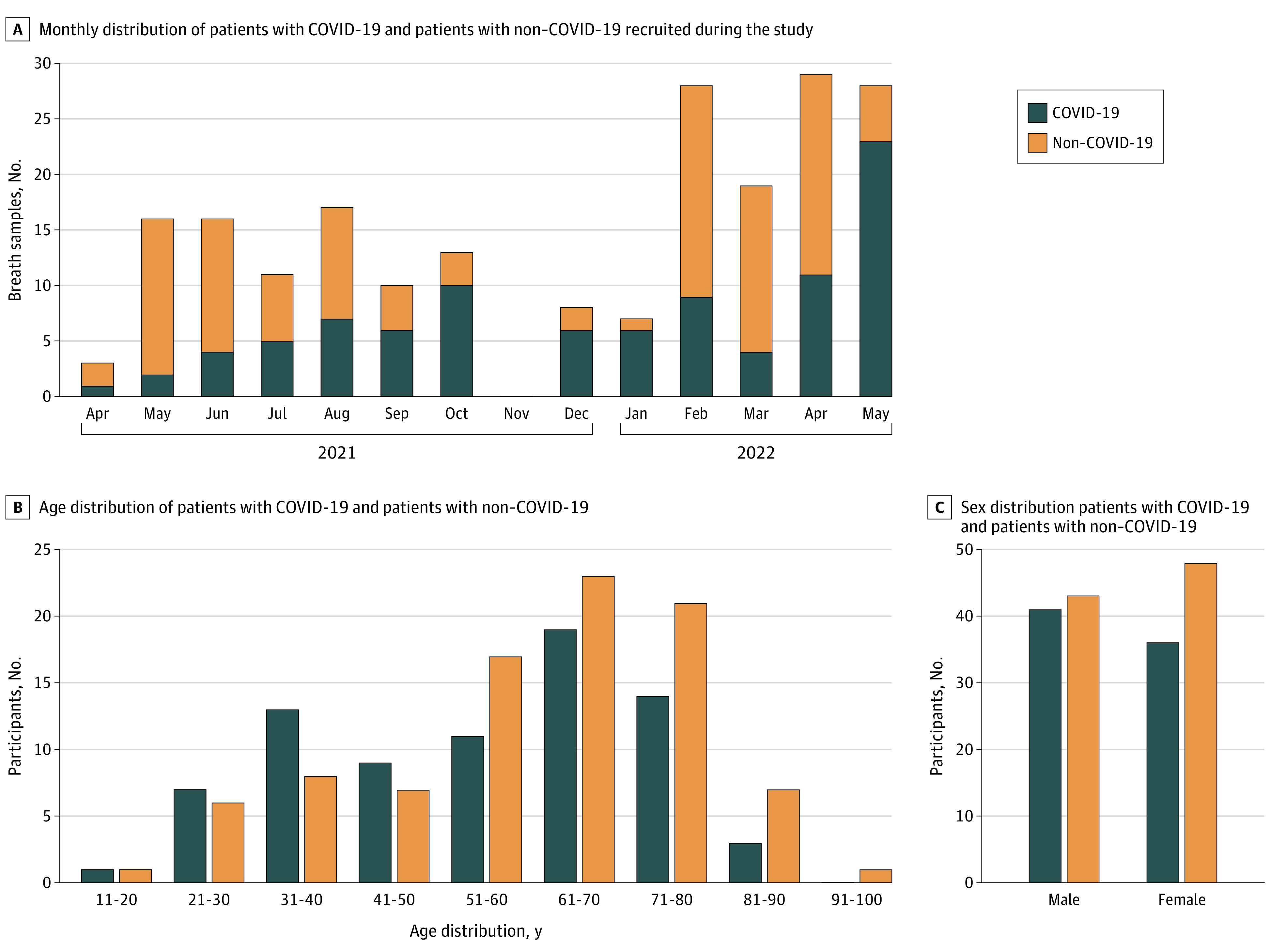
Demographic Information of Study Participants Bar graphs show the distribution of different categories of patients recruited during the study. More information can be found in Table 1 and eFigures 3A and B in Supplement 1.

The demographic details and the total number of participants enrolled in this study are summarized in Figure 1 and Table 1. Overall, a total of 77 patients with COVID-19 (mean [SD] age, 58.5 [16.1] years; 41 [53.2%] male patients; 13 [16.9%] Black and 59 [76.6%] White patients) and 91 patients with non–COVID-19 illness (mean [SD] age, 54.3 [17.1] years; 43 [47.3%] male patients; 11 [12.1%] Black and 76 [83.5%] White patients) were recruited to obtain a total of 205 breath samples. eFigure 4C in Supplement 1 shows the transition from the Delta to Omicron variants in Michigan^41^ between late December 2021 and early January 2022.

**Table 1.** Patients and Breath Samples Used in the Study

Category	COVID-19			Non–COVID-19 illness
	2021^a^	2022^b^	Overall	
Patients, total No. /asymptomatic No.^c^	24/2	53/8	77/10	91/NA
Breath samples, total No. /asymptomatic No.	41/3	53/8	94/11	111/NA
Age, mean (SD), y	NA	NA	58.5 (16.1)	54.3 (17.1)
Sex, No. (%)				
Female	11 (45.8)	25 (47.2)	36 (46.8)	48 (52.7)
Male	13 (54.2)	28 (52.8)	41 (53.2)	43 (47.3)

Abbreviation: NA, not applicable.

^a^ The patients in 2021 were recruited between April 26 and December 14, 2021.

^b^ The patients in 2022 were recruited between January 11 and May 31, 2022.

^c^ The total number of patients in this study is 167. One patient in 2021 originally tested positive for COVID-19 and was later recovered (ie, COVID-19 negative). Therefore, this patient is counted in both COVID-19 and non–COVID-19 illness categories. Overall, 26 patients (11 with COVID-19 and 15 with non–COVID-19 illness) recruited in 2021 were collected and analyzed over multiple days (>24-hour interval) during their stay in the hospital for longitudinal monitoring. Per our institutional review board, we were allowed to collect breath samples up to 10 days from the consent date. Therefore, the number of breath samples is larger than the number of patients in 2021.

### Distinguishing Between COVID-19 (2021) and Non–COVID-19 Illness

**Figure 2.**
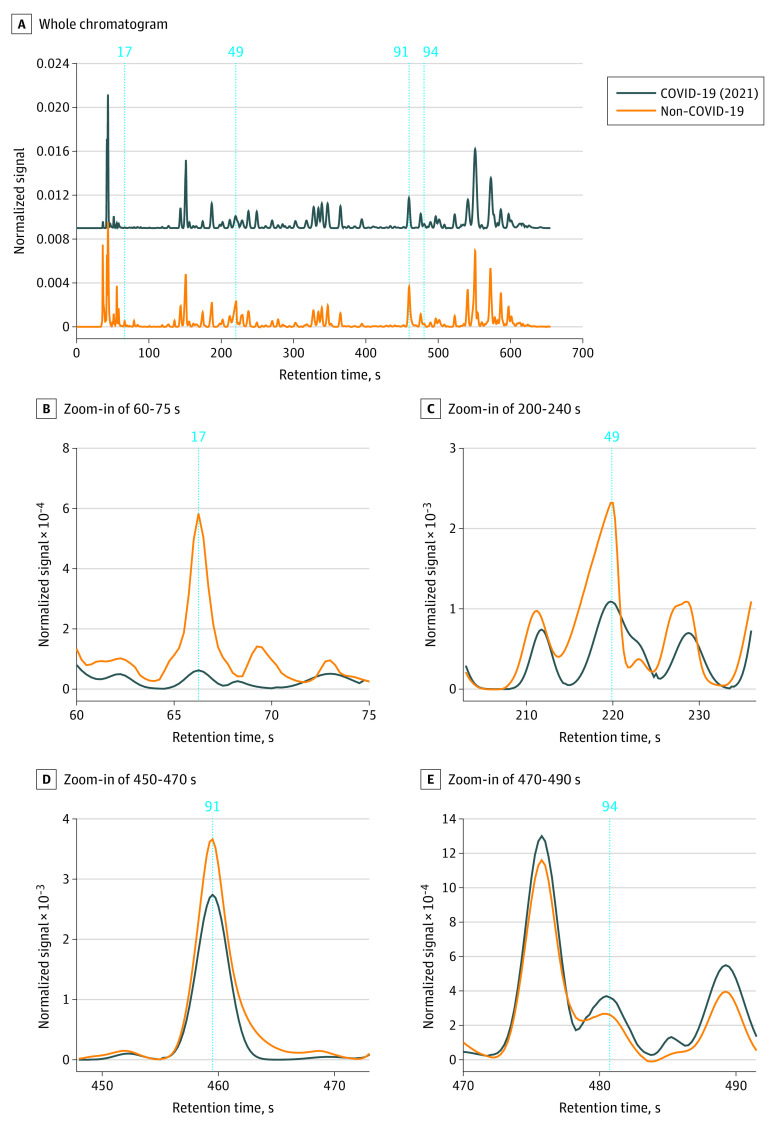
Representative Breath Chromatograms From a Patient With COVID-19 in July 2021 and a Patient with Non–COVID-19 Illness A, Whole chromatograms. The vertical dashed lines and numbers show the positions and listing numbers of the volatile organic compound biomarkers that distinguish between COVID-19 (2021) and non–COVID-19 illness. The violin charts of these VOCs for both COVID-19 (2021) and non–COVID-19 illness are given in eFigure 6 in Supplement 1. B-E, Zoomed-in portions of A. Each chromatogram is normalized to the total area under the curve (from 0 s to 650 s). The COVID-19 (2021) chromatogram is vertically shifted for clarity. COVID-19 (2021) refers to patients who were recruited prior to December 14, 2021, before the SARS-CoV-2 Omicron outbreak in the United States, and were therefore assumed to be infected by Delta or earlier SARS-CoV-2 variants (after April 2021). Patients with non–COVID-19 illness refers to those who were recruited throughout the study (from April 26, 2021, to May 31, 2022).

Figure 2A shows a representative breath chromatogram from a patient with COVID-19 collected in July 2021 and a patient with non–COVID-19 illness. For VOC biomarker discovery, 48 of 152 breath chromatograms were treated as the training set, among which 24 chromatograms were from 24 patients with COVID-19 and 24 chromatograms were from 24 patients with non–COVID-19 illness. The remaining 104 chromatograms (17 from patients with COVID-19 and 87 from patients with non–COVID-19 illness) were used as the testing set.

Four VOCs (peak identification No. 17, 49, 91, and 94) were identified (Figure 2 and Table 2) to distinguish between COVID-19 (2021) and non–COVID-19 illness. A sensitivity of 92.7%, a specificity of 95.5%, a PPV of 88.4%, an NPV of 97.2%, and an accuracy of 94.7% were achieved (Table 3). eFigure 5 in Supplement 1 shows the corresponding PCA plot. In eFigure 5, there were 12 breath samples from 4 patients who had previously tested positive for COVID-19 according to the RT-PCR tests during their stay in the hospital but tested negative when the breath samples were collected and analyzed.

**Table 2.** Volatile Organic Compound Biomarkers Used in the Study

Peak ID	Compound name	Biomarker set^a^
12	Methylcyclopentane	COVID-19 (all variants) vs non–COVID-19 illness
17	Benzene	COVID-19 (2021) vs non–COVID-19 illness; COVID-19 (2022) vs non–COVID-19 illness
27	Unidentified	COVID-19 (2021) vs COVID-19 (2022)
49	Octane	COVID-19 (2021) vs non–COVID-19 illness
67	2,2,4-Trimethylheptane	COVID-19 (all variants) vs non–COVID-19 illness; COVID-19 (2022) vs non–COVID-19 illness; COVID-19 (2021) vs COVID-19 (2022)
69	2,2-Dimethyloctane	COVID-19 (2021) vs COVID-19 (2022)
84	Decene and Dimethyloctadiene (different isomers)	COVID-19 (all variants) vs non–COVID-19 illness
87	Decane	COVID-19 (2022) vs non–COVID-19 illness; COVID-19 (2021) vs COVID-19 (2022)
91	Trimethyloctane (different isomers)	COVID-19 (2021) vs non–COVID-19 illness
94	Methyldecane (different isomers)	COVID-19 (2021) vs non–COVID-19 illness; COVID-19 (2021) vs COVID-19 (2022)
97	Undecane	COVID-19 (2022) vs non–COVID-19 illness
105	Dimethyldecane and Tetramethyloctane (different isomers)	COVID-19 (all variants) vs non–COVID-19 illness

Abbreviation: ID, identification.

^a^ COVID-19 (2021) refers to the patients with COVID-19 who were recruited prior to December 14, 2021, and were therefore assumed to be infected by SARS-CoV-2 Delta or earlier variants. COVID-19 (2022) refers to the patients with COVID-19 who were recruited from January 11 to May 31, 2022, and were therefore assumed to be infected by the SARS-CoV-2 Omicron variant. Non–COVID-19 illness refers to patients with no COVID-19 who were recruited throughout the study (ie, from April 26, 2021, to May 31, 2022). All recovered patients are also included in the non–COVID-19 illness category.

**Table 3.** Statistics Summary^a^

Group	Positive, No.	Negative, No.	Total, No.	Specificity, %	Sensitivity, %	PPV, %	NPV, %	Total accuracy, %
**COVID-19 (2021) vs Non–COVID-19 illness**								
Training								
COVID-19 (2021)	23	1	24	95.8	95.8	95.8	95.8	95.8
Non–COVID-19 illness	1	23	24					
Total	24	24	48					
Testing								
COVID-19 (2021)	15	2	17	95.4	88.2	78.9	97.6	94.2
Non–COVID-19 illness	4	83	87					
Total	19	85	104					
Training and testing								
COVID-19 (2021)	38	3	41	95.5	92.7	88.4	97.2	94.7
Non–COVID-19 illness	5	106	111					
Total	43	109	152					
**COVID-19 (2022) vs Non–COVID-19 illness**								
Training								
COVID-19 (2022)	23	2	25	96.0	92.0	95.8	92.3	94.0
Non–COVID-19 illness	1	24	25					
Total	24	26	50					
Testing								
COVID-19 (2022)	24	4	28	90.7	85.7	75.0	95.1	89.5
Non–COVID-19 illness	8	78	86					
Total	32	82	114					
Training and testing								
COVID-19 (2022)	47	6	53	91.9	88.7	83.9	94.4	90.9
Non–COVID-19 illness	9	102	111					
Total	56	108	164					
**COVID-19 (2021) vs COVID-19 (2022)**								
Training								
COVID-19 (2021)	22	2	24	91.7	91.7	91.7	91.7	91.7
COVID-19 (2022)	2	22	24					
Total	24	24	48					
Testing								
COVID-19 (2021)	16	1	17	89.7	94.1	84.2	96.3	91.3
COVID-19 (2022)	3	26	29					
Total	19	27	46					
Training and testing								
COVID-19 (2021)	38	3	41	90.6	92.7	88.4	94.1	91.5
COVID-19 (2022)	5	48	53					
Total	43	51	94					
**COVID-19 (2021 and 2022) vs Non–COVID-19 illness**								
Training								
COVID-19 (2021 and 2022)	43	5	48	93.8	89.6	93.5	90.0	91.7
Non–COVID-19 illness	3	45	48					
Total	46	50	96					
Testing								
COVID-19 (2021 and 2022)	41	5	46	88.9	89.1	85.4	91.8	89.0
Non–COVID-19 illness	7	56	63					
Total	48	61	108					
Training and testing								
COVID-19 (2021 and 2022)	84	10	94	91.0	89.4	89.4	91.0	90.2
Non–COVID-19 illness	10	101	111					
Total	94	111	205					

^a^ Corresponding 4-fold cross-validation can be found in the eTable in Supplement 1.

### Distinguishing COVID-19 (2022) and Non–COVID-19 Illness

Using the 4 previously identified VOC biomarkers (ie, peak identification No. 17, 49, 91, and 94), breath analyses among patients with COVID-19 (2022) resulted in a substantially lower sensitivity (60.4%) and accuracy (82.1%) but without a change in specificity (95.4%) given that there was no change in the non–COVID-19 illness cases in the testing set given in Table 3. The corresponding PCA plot is provided in eFigure 7 in Supplement 1. This finding suggested a different pathologic response of humans to the Omicron variant vs Delta and earlier variants in 2021. Therefore, a new VOC biomarker search was undertaken in the same manner as for COVID-19 (2021).

For the new VOC biomarker search, 50 of 164 breath chromatograms were treated as the training set, among which 25 chromatograms were from 25 patients with COVID-19 and 25 chromatograms were from 25 patients with non–COVID-19 illness. The remaining 114 chromatograms (28 from patients with COVID-19 and 86 from patients with non–COVID-19 illness) were used as the testing set. A new set of biomarkers (peak identification No. 17, 67, 87, and 97) were identified (Table 2 and eFigure 8 in Supplement 1) to distinguish between COVID-19 (2022) and non–COVID-19 illness. A sensitivity of 88.7%, a specificity of 91.7%, a PPV of 83.9%, an NPV of 94.4%, and an accuracy of 90.9% were achieved (Table 3). eFigure 9 in Supplement 1 shows the corresponding PCA plot.

### Distinguishing COVID-19 (2021) and COVID-19 (2022)

Since the pathology and human response are different between Delta (and earlier variants) and Omicron, we hypothesized that it could be possible to distinguish them using breath analysis. In an additional biomarker search, we used 48 of 94 breath chromatograms as the training set, among which 24 were from 24 patients with COVID-19 (2021) and 24 were from 24 patients with COVID-19 (2022). The remaining 46 chromatograms (17 from patients with COVID-19 [2021] and 29 from patients with COVID-19 [2022]) were used for testing.

A new set of VOC biomarkers (peak identification No. 27, 67, 69, 87, and 94) were identified (Table 2 and eFigure 8 in Supplement 1) to distinguish between presumed Omicron and all previous variants. A sensitivity of 92.7%, a specificity of 90.6%, a PPV of 88.4%, an NPV of 94.1%, and an accuracy of 91.5% were achieved (Table 3). eFigure 10 in Supplement 1 shows the corresponding PCA plot.

### Distinguishing COVID-19 (2021 and 2022) and Non–COVID-19 Illness

An additional question and concern are whether breath analysis can distinguish between patients with COVID-19 and patients with non–COVID-19 illness, regardless of variants. A set of VOC biomarkers that are suitable for all variants (ie, the variants occurring between April 2021 and May 2022, including Delta and Omicron BA.1/BA.2) would be useful for rapid diagnostics without considering the variant. Toward this end, we conducted another biomarker search by using 96 of 205 breath chromatograms as the training set, among which 48 were from 48 patients with COVID-19 (24 from 2021 and 24 from 2022) and 48 were from 48 patients with non–COVID-19 illness. The remaining 109 chromatograms (46 from patients with COVID-19 and 63 from patients with non–COVID-19 illness) were used as the testing set, including those collected in May of 2022.

A new set of VOC biomarkers (peak identification No. 12, 67, 84, and 105) were identified (Table 2 and eFigure 8 in Supplement 1) to distinguish between all variants of COVID-19 occurring between April 2021 and May 2022 and non–COVID-19 illness. A sensitivity of 89.4%, a specificity of 91.0%, a PPV of 89.4%, an NPV of 91.0%, and an accuracy of 90.2% were achieved (Table 3). eFigure 11 in Supplement 1 shows the corresponding PCA plot.

## Discussion

In this study, we demonstrated the ability of a portable GC technology to detect exhaled breath VOC signatures at point of care that were capable of diagnosing COVID-19 with accuracies equivalent to or better than previously reported breath analysis results involving patients in 2020 and 2021 (ie, Delta or earlier variants).^8,9,10,12,13,14,15,17,18,37^ More importantly, we also highlighted the challenge that emerging variants pose to such technologies. On November 30, 2021, Omicron variant B.1.1.529 was determined to be a variant of concern by the United States and since has (with additional subvariants) become the dominant COVID-19 strain in the country. During our study, we noted a significant decrease in sensitivity (from 88.2% to 60.4%) beginning in mid-January 2022. It was during this time that the Omicron variant became the dominant strain. While Michigan Medicine does not do subtyping of the COVID-19 virus, we relied on surveillance and subtyping information provided by Michigan’s Department of Health (eFigure 4C in Supplement 1) and the CDC.^41,42^ Our finding that Omicron seemed to have a different host response than Delta and earlier variants was corroborated by a recent breath analysis study that used a substantially different method.^38^

Since the VOCs detected are likely reflective of inflammation, it is not surprising that changes were noted given that the Delta variant was associated with more severe inflammation.^44,45,46^ As SARS-CoV-2 has mutated over time, these mutations have affected viral characteristics, such as transmissibility and symptom severity. Unlike RT-PCR, which relies on conserved RNA, these mutations have significantly impacted the performance of RATs and other molecular tests.^47^ Other confounding variables that are difficult to account for that could affect breath VOC signatures related to inflammation include changes in the basic biology of SARS-CoV-2, rates of vaccination and boosters, patients’ reinfections, and the advent of outpatient treatments such as paxlovid.

As indicated in the results, a new VOC biomarker search was performed once we noted the drop in performance and the national and regional reporting of the rapid emergence of the Omicron variant, which significantly improved the accuracy of breath analysis. These VOC combinations appear to be able to distinguish patterns different from those observed during the Delta variant peak. When the entire pattern library and models were combined, the overall performance on all participants attained accuracies that could potentially make breath analysis a viable and rapid testing alternative. This becomes of importance during the transition of one variant to another to ensure that declines in diagnostic accuracies are short lived, as variants can emerge at different rates. To implement such a strategy, great vigilance will be needed, as will careful monitoring and communication with health authorities and their subtyping efforts to allow for new VOC signature identification that can then be incorporated into accurate diagnosis regardless of the variant.

While we used RT-PCR as a criterion standard, there continues to be debate regarding its accuracy and concerns over false negatives, which have been reported to be between 1% and 30%.^1,48,49,50^ False negatives can occur for many reasons, ranging from testing that occurs too early or late, poor sample collection, changes in viral shedding, and others. As such, our performance could potentially be better or, with further study, could be used as a confirmatory strategy or as possible adjunct to study and determine the risk of transmissibility or the development of complications linked to inflammation severity. Indeed, we observed 6 participants who tested both positive and negative with RT-PCR within several hours of each other due to duplicative testing orders being placed. These individuals were removed from our analysis.

As demonstrated in our previous longitudinal studies of acute respiratory distress syndrome (ARDS),^22,23^ we were able to note the evolution of severe COVID-19 and its trajectory during the period of the Delta variant. In the current study, we were able to monitor some patients with COVID-19 recruited in 2021 for as many as 10 days. eAppendix 3 in Supplement 1 provides information on 5 patients to highlight the potential of breath analysis in monitoring the trajectories of patients with COVID-19 (some who recovered as well as those who deteriorated) and predicting their clinical outcomes. As such, a potential advantage of breath analysis could be for trajectory monitoring of patients with severe disease resulting in respiratory failure and requiring mechanical ventilation.

During our study, we were also able to capture a subset of patient with asymptomatic COVID-19 and patients with non–COVID-19 illness who were found to be infected by other viruses, such as rhinovirus, human metapneumo virus, human coronavirus OC43 (HCoV-OC43), and enterovirus. The results for those patients are discussed in eAppendix 4 in Supplement 1. The presence of these viruses did not appear to substantially confound the results of GC testing, but further studies are needed.

### Limitations

There are several limitations and challenges to our study and the use of GC as a diagnostic modality in the setting of COVID-19 and other future viral pandemics. We did not perform COVID-19 subtyping but instead relied on our state’s and the CDC’s reports of the incidence of emerging variants.^41,42^ Currently, we are examining our data collected before the Omicron BA.4 and BA.5 variant. We expect to experience decreased accuracy, which may require a new search for diagnostic breath VOC signatures. This, along with issues such as false positives, detection time vs specificity, and personal variations in VOC profiles, represent the major challenges for breath analysis technologies. Additional work is required to identify a universal signature that is perhaps more conserved and less prone to change. Similar to RATs, there will need to be an agreed-upon regulatory approach as to how to track and report the performance of breath diagnostics as variants emerge.

We also do not clearly understand the etiology of the VOCs produced during COVID-19 infection but assume, based on their identification, that they are related to inflammation that may be occurring in both the upper and lower respiratory tract.^22,23,31,51^ The fact that we were able to diagnose COVID-19 in both symptomatic and asymptomatic participants is encouraging. More studies on these particular VOCs, including their origins, may assist in the development of a better understanding of the disease as well as the potential to develop new diagnostics.

Additionally, this was a single-center study using a relatively small patient sample. Larger patient data sets at multiple and diverse clinical locations with details regarding symptom severity, medication use, acute and chronic medical conditions, and other factors will be needed to understand their potential to affect VOC profiles. We also used a limited data science approach to identifying diagnostic VOC patterns. Additional machine learning tools and feature selection techniques such as random forest, neural networks, least absolute shrinkage and selection operator, and minimum redundancy maximum relevance may result in more accurate and stable selection of VOCs and better diagnostic performance.

## Conclusions

The findings of this diagnostic study suggest that breath analysis using point-of-care GC may be a promising modality for detecting COVID-19 and similar diseases that result in VOC production. However, similar to other diagnostic modalities, such as RAT, challenges are posed by the dynamic emergence of viral variants. The results of this study warrant additional investment and evaluation of how to overcome these challenges and to use breath analysis to improve the diagnosis and care of patients.

## Notes

Supplement 1. **eAppendix 1.** Breath Sampling, GC Operation, and Characterization **eAppendix 2.** GC-MS Identification of Breath Biomarkers **eAppendix 3.** Trajectory Monitoring of Patients with COVID-19 (2021) **eAppendix 4.** Asymptomatic Patients and Cross-Reactivity Examination **eFigure 1.** Breath Collection From Ventilated and Nonventilated Patients **eFigure 2.** Inter-GC Characterization of 5 Portable GC Devices Used in the Study With the Same Breath Sample Collected From a Patient With Non–COVID-19 **eFigure 3.** Setup of Portable GC to MS and Chromatograms Obtained Concomitantly by the Photoionization Detector (PID) in GC and MS for the Same Breath Sample **eFigure 4.** Demographic Information of the Human Participants in the Study **eFigure 5.** PCA Plot Using the 4 VOC Biomarkers in Figure 2 and Table 2 (in the Main Text) to Distinguish Between COVID-19 (2021) and Non–COVID-19 **eFigure 6.** Violin Charts of the 4 Biomarkers Used in Figure 2 (in the Main Text) to Distinguish Between COVID-19 (2021) and Non–COVID-19 **eFigure 7.** PCA Plot for Patient With COVID-19 (2022) When the 4 VOC Biomarkers in Figure 2 and Table 2 (in the Main Text) for COVID-19 (2021) Are Used **eFigure 8.** Positions of the Biomarkers Used in eFigure 9 to Distinguish Between COVID-19 (2022) and Non–COVID-19 (Peak ID: 17, 67, 87, 97), in eFigure 10 to Distinguish Between COVID-19 (2021) and COVID19 (2022) (Peak ID: 27, 67, 69, 87, 94), and in eFigure 11 to Distinguish Between COVID19 (All Variants Occurring Between April 2021 and May 2022) and Non–COVID-19 (Peak ID: 12, 67, 84, 105) **eFigure 9.** PCA Plot Using 4 VOC Biomarkers in eFigure 8 and Table 2 (in the Main Text) to Distinguish Between COVID-19 (2022) and Non–COVID-19 **eFigure 10.** PCA Plot Using 5 VOC Biomarkers in eFigure 8 and Table 2 (in the Main Text) to Distinguish Between COVID-19 Omicron and Previous Variants **eFigure 11.** PCA Plot Using 4 VOC Biomarkers in eFigure 8 and Table 2 (in the Main Text) to Distinguish Between COVID-19 (All Variants) and Non–COVID-19 **eFigure 12.** Trajectories on the PCA Plot for Various Patients Monitored for Multiple Days **eFigure 13.** Patients With Asymptomatic COVID-19 (2021) and Patients Who Were Infected by Other Viruses on the Same PCA Plot as in eFigure 5 **eFigure 14.** Patients with Asymptomatic COVID-19 (2022) and Patients Who Were Infected by Other Viruses on the Same PCA Plot as eFigure 9 **eFigure 15.** Patients with Asymptomatic COVID-19 (Regardless of Variants) and Patients With Non–COVID-19 Who Were Infected by Other Viruses on the same PCA Plot as eFigure 11 **eTable.** Four-Fold Cross-Validation
eReferences.

Supplement 2.
Data Sharing Statement

## References

[1] Osterman A, Badell I, Basara E, . Impaired detection of Omicron by SARS-CoV-2 rapid antigen tests. Med Microbiol Immunol. 2022;211(2-3):105-117. doi:10.1007/s00430-022-00730-z 35187580 PMC8858605

[2] Hardick J, Gallagher N, Sachithanandham J, . Evaluation of four point of care (POC) antigen assays for the detection of the SARS-CoV-2 variant Omicron. Microbiol Spectr. 2022;10(3):e0102522. doi:10.1128/spectrum.01025-22 35616382 PMC9241858

[3] Stanley S, Hamel DJ, Wolf ID, . Limit of detection for rapid antigen testing of the SARS-CoV-2 Omicron and Delta variants of concern using live-virus culture. J Clin Microbiol. 2022;60(5):e0014022. doi:10.1128/jcm.00140-22 35440165 PMC9116160

[4] Bayart J-L, Degosserie J, Favresse J, . Analytical sensitivity of six SARS-CoV-2 Rapid antigen tests for Omicron versus Delta variant. Viruses. 2022;14(4):654. doi:10.3390/v14040654 35458384 PMC9031584

[5] Lamote K, Janssens E, Schillebeeckx E, Lapperre TS, De Winter BY, van Meerbeeck JP. The scent of COVID-19: viral (semi-)volatiles as fast diagnostic biomarkers? J Breath Res. 2020;14(4):042001. doi:10.1088/1752-7163/aba105 32599571

[6] Davis CE, Schivo M, Kenyon NJ. A breath of fresh air—the potential for COVID-19 breath diagnostics. EBioMedicine. 2021;63:103183. doi:10.1016/j.ebiom.2020.103183 33418507 PMC7785417

[7] Giovannini G, Haick H, Garoli D. Detecting COVID-19 from breath: a game changer for a big challenge. ACS Sens. 2021;6(4):1408-1417. doi:10.1021/acssensors.1c00312 33825440 PMC8043202

[8] Shan B, Broza YY, Li W, . Multiplexed nanomaterial-based sensor array for detection of COVID-19 in exhaled breath. ACS Nano. 2020;14(9):12125-12132. doi:10.1021/acsnano.0c05657 32808759

[9] Ruszkiewicz DM, Sanders D, O’Brien R, . Diagnosis of COVID-19 by analysis of breath with gas chromatography-ion mobility spectrometry—a feasibility study. EClinicalMedicine. 2020;29:100609. doi:10.1016/j.eclinm.2020.100609 33134902 PMC7585499

[10] Chen H, Qi X, Zhang L, . COVID-19 screening using breath-borne volatile organic compounds. J Breath Res. 2021;15(4):047104. doi:10.1088/1752-7163/ac2e57 34624875

[11] Steppert C, Steppert I, Sterlacci W, Bollinger T. Rapid detection of SARS-CoV-2 infection by multicapillary column coupled ion mobility spectrometry (MCC-IMS) of breath: a proof of concept study. J Breath Res. 2021;15(2):027105. doi:10.1088/1752-7163/abe5ca 33578396

[12] Grassin-Delyle S, Roquencourt C, Moine P, ; Garches COVID-19 Collaborative Group RECORDS Collaborators and Exhalomics Collaborators. Metabolomics of exhaled breath in critically ill COVID-19 patients: a pilot study. EBioMedicine. 2021;63:103154. doi:10.1016/j.ebiom.2020.103154 33279860 PMC7714658

[13] Ibrahim W, Cordell RL, Wilde MJ, . Diagnosis of COVID-19 by exhaled breath analysis using gas chromatography-mass spectrometry. ERJ Open Res. 2021;7(3):00139-02021. doi:10.1183/23120541.00139-2021 34235208 PMC8255539

[14] Wintjens AGWE, Hintzen KFH, Engelen SME, . Applying the electronic nose for pre-operative SARS-CoV-2 screening. Surg Endosc. 2021;35(12):6671-6678. doi:10.1007/s00464-020-08169-0 33269428 PMC7709806

[15] Exline MC, Stanacevic M, Bowman AS, Gouma P-I. Exhaled nitric oxide detection for diagnosis of COVID-19 in critically ill patients. PLoS One. 2021;16(10):e0257644. doi:10.1371/journal.pone.0257644 34710098 PMC8553051

[16] Berna AZ, Akaho EH, Harris RM, . Reproducible breath metabolite changes in children with SARS-CoV-2 infection. ACS Infect Dis. 2021;7(9):2596-2603. doi:10.1021/acsinfecdis.1c00248 34319698 PMC8353987

[17] Shlomo IB, Frankenthal H, Laor A, Greenhut AK. Detection of SARS-CoV-2 infection by exhaled breath spectral analysis: introducing a ready-to-use point-of-care mass screening method. EClinicalMedicine. 2022;45:101308. doi:10.1016/j.eclinm.2022.101308 35224472 PMC8856887

[18] Woollam M, Angarita-Rivera P, Siegel AP, Kalra V, Kapoor R, Agarwal M. Exhaled VOCs can discriminate subjects with COVID-19 from healthy controls. J Breath Res. 2022;16(3):036002. doi:10.1088/1752-7163/ac696a 35453137

[19] Subali AD, Wiyono L, Yusuf M, Zaky MFA. The potential of volatile organic compounds-based breath analysis for COVID-19 screening: a systematic review & meta-analysis. Diagn Microbiol Infect Dis. 2022;102(2):115589. doi:10.1016/j.diagmicrobio.2021.115589 34879323 PMC8556067

[20] Nurputra DK, Kusumaatmaja A, Hakim MS, . Fast and noninvasive electronic nose for sniffing out COVID-19 based on exhaled breath-print recognition. NPJ Digit Med. 2022;5(1):115. doi:10.1038/s41746-022-00661-2 35974062 PMC9379872

[21] Sharma R, Zang W, Zhou M, . Real time breath analysis using portable gas chromatography for adult asthma phenotypes. Metabolites. 2021;11(5):265. doi:10.3390/metabo11050265 33922762 PMC8145057

[22] Sharma R, Zhou M, Tiba MH, . Breath analysis for detection and trajectory monitoring of acute respiratory distress syndrome in swine. ERJ Open Res. 2022;8(1):00154-02021. doi:10.1183/23120541.00154-2021 35174248 PMC8841990

[23] Zhou M, Sharma R, Zhu H, . Rapid breath analysis for acute respiratory distress syndrome diagnostics using a portable two-dimensional gas chromatography device. Anal Bioanal Chem. 2019;411(24):6435-6447. doi:10.1007/s00216-019-02024-5 31367803 PMC6722019

[24] Hashoul D, Haick H. Sensors for detecting pulmonary diseases from exhaled breath. Eur Respir Rev. 2019;28(152):190011. doi:10.1183/16000617.0011-2019 31243097 PMC9489036

[25] van de Kant KDG, van der Sande LJTM, Jöbsis Q, van Schayck OCP, Dompeling E. Clinical use of exhaled volatile organic compounds in pulmonary diseases: a systematic review. Respir Res. 2012;13(1):117. doi:10.1186/1465-9921-13-117 23259710 PMC3549749

[26] Neerincx AH, Geurts BP, Habets MFJ, . Identification of *Pseudomonas aeruginosa* and *Aspergillus fumigatus* mono- and co-cultures based on volatile biomarker combinations. J Breath Res. 2016;10(1):016002. doi:10.1088/1752-7155/10/1/016002 26824272

[27] van Geffen WH, Bruins M, Kerstjens HAM. Diagnosing viral and bacterial respiratory infections in acute COPD exacerbations by an electronic nose: a pilot study. J Breath Res. 2016;10(3):036001. doi:10.1088/1752-7155/10/3/036001 27310311

[28] Schubert JK, Müller WP, Benzing A, Geiger K. Application of a new method for analysis of exhaled gas in critically ill patients. Intensive Care Med. 1998;24(5):415-421. doi:10.1007/s001340050589 9660254

[29] van Oort PMP, de Bruin S, Weda H, Knobel HH, Schultz MJ, Lieuwe D; Mars Consortium. Exhaled breath metabolomics for the diagnosis of pneumonia in intubated and mechanically-ventilated intensive care unit (ICU)-patients. Int J Mol Sci. 2017;18(2):449. doi:10.3390/ijms18020449 28218729 PMC5343983

[30] Bos LDJ, Schultz MJ, Sterk PJ. Exhaled breath profiling for diagnosing acute respiratory distress syndrome. BMC Pulm Med. 2014;14:72. doi:10.1186/1471-2466-14-72 24767549 PMC4021554

[31] Bos LDJ, Weda H, Wang Y, . Exhaled breath metabolomics as a noninvasive diagnostic tool for acute respiratory distress syndrome. Eur Respir J. 2014;44(1):188-197. doi:10.1183/09031936.00005614 24743964

[32] Schnabel R, Fijten R, Smolinska A, . Analysis of volatile organic compounds in exhaled breath to diagnose ventilator-associated pneumonia. Sci Rep. 2015;5:17179. doi:10.1038/srep17179 26608483 PMC4660425

[33] Filipiak W, Beer R, Sponring A, . Breath analysis for in vivo detection of pathogens related to ventilator-associated pneumonia in intensive care patients: a prospective pilot study. J Breath Res. 2015;9(1):016004. doi:10.1088/1752-7155/9/1/016004 25557917

[34] Amann A, Costello BdeL, Miekisch W, . The human volatilome: volatile organic compounds (VOCs) in exhaled breath, skin emanations, urine, feces and saliva. J Breath Res. 2014;8(3):034001. doi:10.1088/1752-7155/8/3/034001 24946087

[35] de Lacy Costello B, Amann A, Al-Kateb H, . A review of the volatiles from the healthy human body. J Breath Res. 2014;8(1):014001. doi:10.1088/1752-7155/8/1/014001 24421258

[36] Sharma R, Zhou M, Hunter MD, Fan X. Rapid in situ analysis of plant emission for disease diagnosis using a portable gas chromatography device. J Agric Food Chem. 2019;67(26):7530-7537. doi:10.1021/acs.jafc.9b02500 31184878

[37] US Food and Drug Association. InspectIR COVID-19 breathalyzer. April 14, 2022. Accessed January 24, 2023. https://www.fda.gov/media/157723/download

[38] McCartney MM, Borras E, Rojas DE, . Predominant SARS-CoV-2 variant impacts accuracy when screening for infection using exhaled breath vapor. Commun Med (Lond). 2022;2(1):158. doi:10.1038/s43856-022-00221-5 36482179 PMC9731983

[39] Department of Health and Human Services. Overview information. Accessed January 24, 2023. https://grants.nih.gov/grants/guide/rfa-files/rfa-od-20-017.html

[40] Gillies CE, Jennaro TS, Puskarich MA, . A multilevel bayesian approach to improve effect size estimation in regression modeling of metabolomics data utilizing imputation with uncertainty. Metabolites. 2020;10(8):319. doi:10.3390/metabo10080319 32781624 PMC7465156

[41] MI COVID response data and modeling update. June 21, 2022. Accessed January 24, 2023. https://www.michigan.gov/coronavirus/-/media/Project/Websites/coronavirus/Michigan-Data/Data-and-Modeling-Updates/20220621-Data-and-modeling-update_vFINAL.pdf?rev=e6f902ee8e8d4a969336c1bc1a049008&hash=5FF667FAEE5F5A175CA304F1FC125CB7

[42] US Centers for Disease Control and Prevention. COVID data tracker. Accessed January 24, 2023. https://covid.cdc.gov/covid-data-tracker/#datatracker-home

[43] Michigan Data. Coronavirus. Accessed January 24, 2023. https://www.michigan.gov/coronavirus/stats

[44] Hu K, Lin L, Liang Y, . COVID-19: risk factors for severe cases of the Delta variant. Aging (Albany NY). 2021;13(20):23459-23470. doi:10.18632/aging.203655 34710058 PMC8580340

[45] Liu X, Mostafavi H, Ng WH, . The Delta SARS-CoV-2 variant of concern induces distinct pathogenic patterns of respiratory disease in K18-hACE2 transgenic mice compared to the ancestral strain from Wuhan. mBio. 2022;13(3):e0068322. doi:10.1128/mbio.00683-22 35420469 PMC9239116

[46] Luo CH, Morris CP, Sachithanandham J, . Infection with the SARS-CoV-2 Delta variant is associated with higher infectious virus loads compared to the Alpha Variant in both unvaccinated and vaccinated individuals. medRxiv. Preprint posted online August 20, 2021. doi:10.1101/2021.08.15.21262077

[47] US Food and Drug Administration. SARS-CoV-2 viral mutations: impact on COVID-19 tests. Accessed January 24, 2023. https://www.fda.gov/medical-devices/coronavirus-covid-19-and-medical-devices/sars-cov-2-viral-mutations-impact-covid-19-tests

[48] Arevalo-Rodriguez I, Buitrago-Garcia D, Simancas-Racines D, . False-negative results of initial RT-PCR assays for COVID-19: a systematic review. PLoS One. 2020;15(12):e0242958. doi:10.1371/journal.pone.0242958 33301459 PMC7728293

[49] Kanji JN, Zelyas N, MacDonald C, . False negative rate of COVID-19 PCR testing: a discordant testing analysis. Virol J. 2021;18(1):13. doi:10.1186/s12985-021-01489-0 33422083 PMC7794619

[50] Pecoraro V, Negro A, Pirotti T, Trenti T. Estimate false-negative RT-PCR rates for SARS-CoV-2: a systematic review and meta-analysis. Eur J Clin Invest. 2022;52(2):e13706. doi:10.1111/eci.13706 34741305 PMC8646643

[51] Bos LDJ. Diagnosis of acute respiratory distress syndrome by exhaled breath analysis. Ann Transl Med. 2018;6(2):33. doi:10.21037/atm.2018.01.17 29430450 PMC5799150

