# Supplementary material for: Portable Breath-Based Volatile Organic Compound Monitoring for the Detection of COVID-19 During the Circulation of the SARS-CoV-2 Delta Variant and the Transition to the SARS-CoV-2 Omicron Variant
Source: JAMA Netw Open. 2023 Feb 28;6(2):e230982. doi: 10.1001/jamanetworkopen.2023.0982 (PMC9975913; doi:10.1001/jamanetworkopen.2023.0982)
Supplement: Supplement 1. — eAppendix 1. Breath Sampling, GC Operation, and Characterization eAppendix 2. GC-MS Identification of Breath Biomarkers eAppendix 3. Trajectory Monitoring of Patients with COVID-19 (2021) eAppendix 4. Asymptomatic Patients and Cross-Reactivity Examination eFigure 1. Breath Collection From Ventilated and Nonventilated Patients eFigure 2. Inter-GC Characterization of 5 Portable GC Devices Used in the Study With the Same Breath Sample Collected From a Patient With Non–COVID-19 eFigure 3. Setup of Portable GC to MS and Chromatograms Obtained Concomitantly by the Photoionization Detector (PID) in GC and MS for the Same Breath Sample eFigure 4. Demographic Information of the Human Participants in the Study eFigure 5. PCA Plot Using the 4 VOC Biomarkers in Figure 2 and Table 2 (in the Main Text) to Distinguish Between COVID-19 (2021) and Non–COVID-19 eFigure 6. Violin Charts of the 4 Biomarkers Used in Figure 2 (in the Main Text) to Distinguish Between COVID-19 (2021) and Non–COVID-19 eFigure 7. PCA Plot for Patient With COVID-19 (2022) When the 4 VOC Biomarkers in Figure 2 and Table 2 (in the Main Text) for COVID-19 (2021) Are Used eFigure 8. Positions of the Biomarkers Used in eFigure 9 to Distinguish Between COVID-19 (2022) and Non–COVID-19 (Peak ID: 17, 67, 87, 97), in eFigure 10 to Distinguish Between COVID-19 (2021) and COVID19 (2022) (Peak ID: 27, 67, 69, 87, 94), and in eFigure 11 to Distinguish Between COVID19 (All Variants Occurring Between April 2021 and May 2022) and Non–COVID-19 (Peak ID: 12, 67, 84, 105) eFigure 9. PCA Plot Using 4 VOC Biomarkers in eFigure 8 and Table 2 (in the Main Text) to Distinguish Between COVID-19 (2022) and Non–COVID-19 eFigure 10. PCA Plot Using 5 VOC Biomarkers in eFigure 8 and Table 2 (in the Main Text) to Distinguish Between COVID-19 Omicron and Previous Variants eFigure 11. PCA Plot Using 4 VOC Biomarkers in eFigure 8 and Table 2 (in the Main Text) to Distinguish Between COVID-19 (All Variants) and Non–COVID-19 eFigure 12. Trajec [file jamanetwopen-e230982-s001.pdf]

Supplemental Online Content

Sharma R, Zang W, Tabartehfarahani A, et al. Portable breath-based volatile organic compound monitoring for the detection of COVID-19 during the circulation of the SARS-CoV-2 Delta variant and the transition to the SARS-CoV-2 Omicron variant. *JAMA Netw Open.* 2023;6(2):e230982. doi:10.1001/jamanetworkopen.2023.0982

eAppendix 1. Breath Sampling, GC Operation, and Characterization

eAppendix 2. GC-MS Identification of Breath Biomarkers

eAppendix 3. Trajectory Monitoring of Patients with COVID-19 (2021)

eAppendix 4. Asymptomatic Patients and Cross-Reactivity Examination

eFigure 1. Breath Collection From Ventilated and Nonventilated Patients

eFigure 2. Inter-GC Characterization of 5 Portable GC Devices Used in the Study With the Same Breath Sample Collected From a Patient With Non–COVID-19

eFigure 3. Setup of Portable GC to MS and Chromatograms Obtained Concomitantly by the Photoionization Detector (PID) in GC and MS for the Same Breath Sample

eFigure 4. Demographic Information of the Human Participants in the Study

eFigure 5. PCA Plot Using the 4 VOC Biomarkers in Figure 2 and Table 2 (in the Main Text) to Distinguish Between COVID-19 (2021) and Non–COVID-19

eFigure 6. Violin Charts of the 4 Biomarkers Used in Figure 2 (in the Main Text) to Distinguish Between COVID-19 (2021) and Non–COVID-19

eFigure 7. PCA Plot for Patient With COVID-19 (2022) When the 4 VOC Biomarkers in Figure 2 and Table 2 (in the Main Text) for COVID-19 (2021) Are Used

eFigure 8. Positions of the Biomarkers Used in eFigure 9 to Distinguish Between COVID-19 (2022) and Non–COVID-19 (Peak ID: 17, 67, 87, 97), in eFigure 10 to Distinguish Between COVID-19 (2021) and COVID19 (2022) (Peak ID: 27, 67, 69, 87, 94), and in eFigure 11 to Distinguish Between COVID19 (All Variants Occurring Between April 2021 and May 2022) and Non–COVID-19 (Peak ID: 12, 67, 84, 105)

eFigure 9. PCA Plot Using 4 VOC Biomarkers in eFigure 8 and Table 2 (in the Main Text) to Distinguish Between COVID-19 (2022) and Non–COVID-19

eFigure 10. PCA Plot Using 5 VOC Biomarkers in eFigure 8 and Table 2 (in the Main Text) to Distinguish Between COVID-19 Omicron and Previous Variants

eFigure 11. PCA Plot Using 4 VOC Biomarkers in eFigure 8 and Table 2 (in the Main Text) to Distinguish Between COVID-19 (All Variants) and Non–COVID-19

eFigure 12. Trajectories on the PCA Plot for Various Patients Monitored for Multiple Days

eFigure 13. Patients With Asymptomatic COVID-19 (2021) and Patients Who Were Infected by Other Viruses on the Same PCA Plot as in eFigure 5

© 2023 Sharma R et al. *JAMA Network Open.*

eFigure 14. Patients with Asymptomatic COVID-19 (2022) and Patients Who Were Infected by Other Viruses on the Same PCA Plot as eFigure 9

eFigure 15. Patients with Asymptomatic COVID-19 (Regardless of Variants) and Patients With Non–COVID-19 Who Were Infected by Other Viruses on the same PCA Plot as eFigure 11

eTable. Four-Fold Cross-Validation

eReferences.

This supplemental material has been provided by the authors to give readers additional information about their work.

eAppendix 1. Breath Sampling, GC Operation, and Characterization

eA1.1. Breath sampling

The breath sample was collected into a 5 L Tedlar bag as shown in eFigure 1.

eA1.2. GC operation

The details about the portable GC itself can be found in our previous study[1]. The operation of the portable GC is described briefly:

1. Connection of the Tedlar bag to the GC inlet;
2. Withdrawal of the breath sample into the GC device by GC internal pump at a flow rate of 70 mL/min for 5 minutes.
3. GC separation of the breath sample
4. GC self-cleaning

The total time for Steps 1-4 is ~25 minutes.

eA1.3. Inter-GC characterization

Five portable GC devices were constructed for this study. To evaluate the repeatability, the same breath sample collected from a non-COVID subject was respectively analyzed by those GC devices. In addition, a chromatogram of normal alkanes mixtures, C_6-C_{11}, is obtained from one of the five GC devices operated under identical conditions (such as temperature ramping and flow rate, *etc*.) to mark the breath VOC peak positions against those of C_6-C_{11} (eFigure 2). It is seen that all breath VOC peaks are aligned well (within +/- 0.5 seconds) after correlation optimized warping algorithms, which allows us to pinpoint the peaks among the chromatograms obtained from different GC devices by using their respective retention times. All the chromatograms (and the subsequent processed data) in the study were obtained from those five GC devices. In our data analysis, we treat all data equally regardless of the GC device used to obtain them.

For each individual breath sample, the number of chromatographic peaks range is approximately 90. The total number of chromatographic peaks among all patients who we recruited in the study is 131. We label them from Peak 1 to Peak 131 from the earliest peak to the latest peak.

© 2023 Sharma R et al. *JAMA Network Open.*

eAppendix 2. GC-MS Identification of Breath Biomarkers

To chemically identify the breath biomarkers, the outlet of our portable GC was connected to an Agilent mass spectrometry (MS), as illustrated in eFigure 3(A), which allows us to compare the chromatograms obtained concomitantly by the photoionization detector (PID) in our GC and MS for the same breath sample (see eFigure 3(B)).

GC-MS is gold-standard for VOC identification in complex matrices, but this method can fail when the obtained data are contaminated with additional molecule fragments due to coelution. These additional fragments can lead to VOC misidentification by automated MS software, due to the reduced the spectrum match factor below the identification threshold and the presence of contaminated fragments. In order to address this problem, we developed the following pipeline for more accurate identification of breath biomarkers.

1. Use the NIST MS library for chemical identification. The NIST MS library often provides many possible “hits”.
2. The MS spectrum and mass fragments are manually checked to confirm the presence of the main mass fragments in the NIST suggested compound by MS library.
3. Check whether the suggested compound in Step 1 is present in human breath from previous studies. Particularly, the website maintained by EPA (https://comptox.epa.gov/dashboard/chemical_lists/VOLATILOME) is adopted, which provides a list of 1117 compounds (as of November 2021) in human breath.
4. Further narrow down the compound candidates by comparing their vapor pressure (or boiling point) with their neighboring normal alkanes (eFigure 2).

The final identification of the breath biomarkers is listed in Table 2 in the main text.

eAppendix 3. Trajectory Monitoring of Patients with COVID-19 (2021)

One of the prominent advantages of breath analysis is its ability of continuous and non-invasive monitoring of patients, as demonstrated in our previous studies[1-3]. In the current study, some COVID-19 positive patients (all recruited in 2021) were monitored for up to 10 days since their recruitment into the study. Below we present 5 cases to highlight the potential of breath analysis in monitoring COVID-19 patients' trajectories and predicting their clinical outcomes. Such ability was also demonstrated in our previous studies in acute respiratory distress syndrome in both human and swine[2,3].

Recovery cases. eFigures 12(A) and (B) show the trajectories of four COVID-19 patients (Patients *a*, *b*, *c*, and *d*). All the patients were initially COVID-19 positive, and later recovered and were discharged from the hospital.

Patient *a* was sampled for 6 days (Day 2, 3, 4, 9, and 10). Until Day 4, the patient was COVID-19 positive. On the 9th day the patient was listed as non-COVID-19 based on the RT-PCR within 18 hours of breath analysis. Later, this patient was extubated and discharged from the hospital, approximately 2 months after our last day of breath analysis.

Patient *b* on Day 2 (data point marked as *b*2) represents the most severe case that we enrolled among the entire patient pool, whose data point is the farthest in the distance from the boundary (grey line in the PCA plot). As per our GC measurement, this patient showed recovery (moving towards the gray boundary line) as we monitored the patient breath on Day 2, 4, and 8. We could not continue our measurement further after the patient consent expired, but later this patient recovered and was discharged from the hospital 15 days after our last breath analysis. We observed a similar trajectory with Patients *c* and *d*. Their breath measurements showed the recovery trend (moving towards the gray boundary line); later those two patients recovered and were discharged from the hospital after last breath measurement.

Deterioration case. Patient *e* was COVID-19 positive, and his/her situation deteriorated over time. This patient died 21 days after our last breath measurement (on Day 10). The worsened case was corroborated by our multiple-day breath measurements as in eFigure 12(C) (the data point moving farther away from the gray boundary line on the PCA plot).

© 2023 Sharma R et al. *JAMA Network Open.*

eAppendix 4. Asymptomatic Patients and Cross-Reactivity Examination

During our study, we also closely monitored the asymptomatic COVID-19 patients and non-COVID-19 patients infected by other viruses such as rhinovirus, human metapneumo virus, HCoV-OC43, and enterovirus. For better clarity, their breath analysis data are re-plotted in eFigures 13-15 using the same PCA plot as in eFigures 5, 9, and 11, respectively.

Below we provide a list of symptoms Michigan Medicine uses to screen symptomatic and asymptomatic COVID-19 patients. (1) We first examined the COVID-19 RT-PCR test; (2) then examined the upper respiratory infection (URI) symptoms (cough, sore throat, and shortness of breath); (3) looked for other signs known to be related to COVID-19 such as abdominal pain, nausea, vomiting, diarrhea, body aches, loss of taste or smell, fever, chills, and fatigue, *etc*.; (4) followed by patients with concerns for various infections.

Asymptomatic patients were categorized by finding a COVID-19 positive test but without symptoms above. If a patient was not admitted to the hospital with URI symptoms, the chief complaint was not COVID-19 related, and there was a lack of symptoms, this patient was also counted as asymptomatic.

© 2023 Sharma R et al. *JAMA Network Open.*

eFigure 1. Breath Collection From Ventilated and Nonventilated Patients

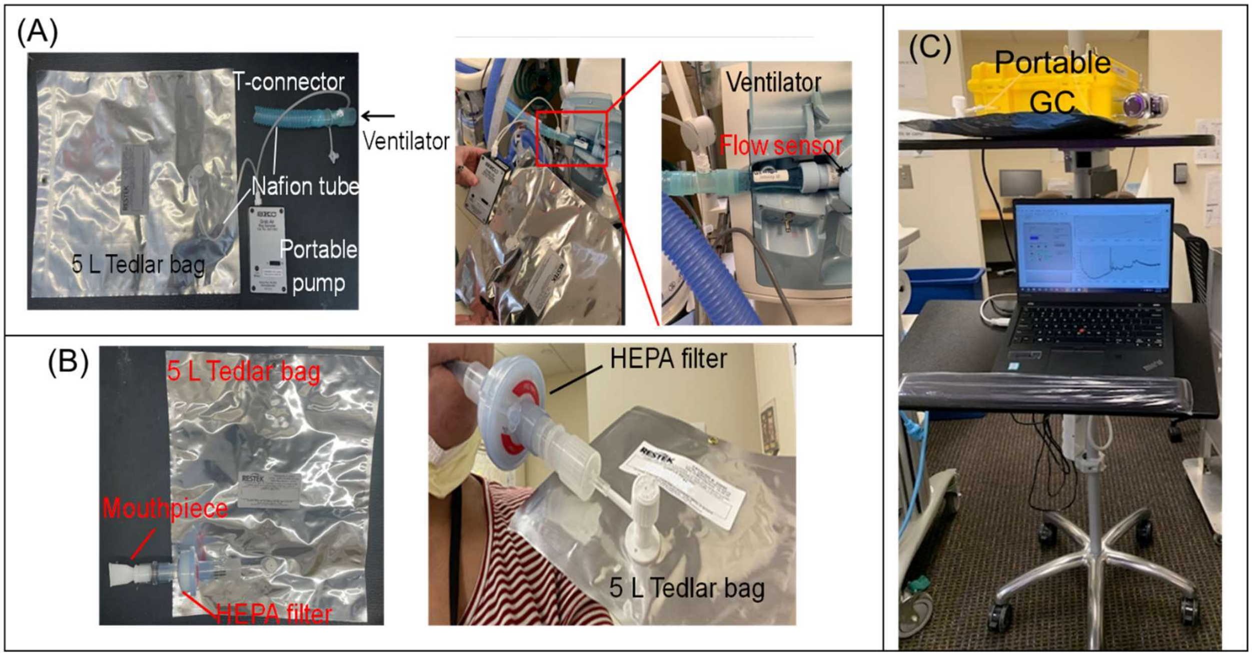

eFigure 1. (A) Breath collection from a ventilated patient. A portable pump was used to pump breath into a 5 L Tedlar bag via a T-connector connected to the flow sensor on the ventilator expiratory port. (B) Breath collection from a non-ventilated patient into a 5 L Tedlar bag via a mouthpiece and an in-line HEPA filter. (C) Photo showing a portable GC (housed in a yellow box) secured on a mobile cart, as well as a tethered laptop for on-board control.

© 2023 Sharma R et al. *JAMA Network Open.*

eFigure 2. Inter-GC Characterization of 5 Portable GC Devices Used in the Study With the Same Breath Sample Collected From a Patient With Non–COVID-19

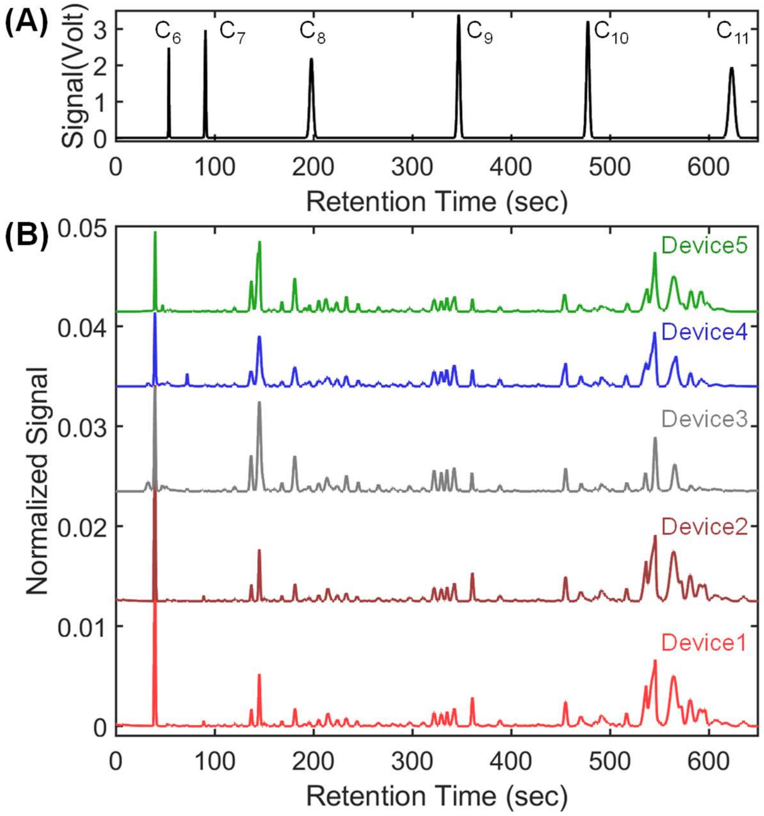

eFigure 2. Inter-GC characterization of five portable GC devices used in the study with the same breath sample collected from a non-COVID-19 subject. Note that the chromatogram of C_6-C_{11} (A) obtained from one of the five GC devices operated under identical conditions (such as temperature ramping, and flow rate, *etc.*) is superimposed on those human breath chromatograms (B) to mark the breath VOC peaks against those of C_6-C_{11}.

© 2023 Sharma R et al. *JAMA Network Open.*

eFigure 3. Setup of Portable GC to MS and Chromatograms Obtained Concomitantly by the Photoionization Detector (PID) in GC and MS for the Same Breath Sample

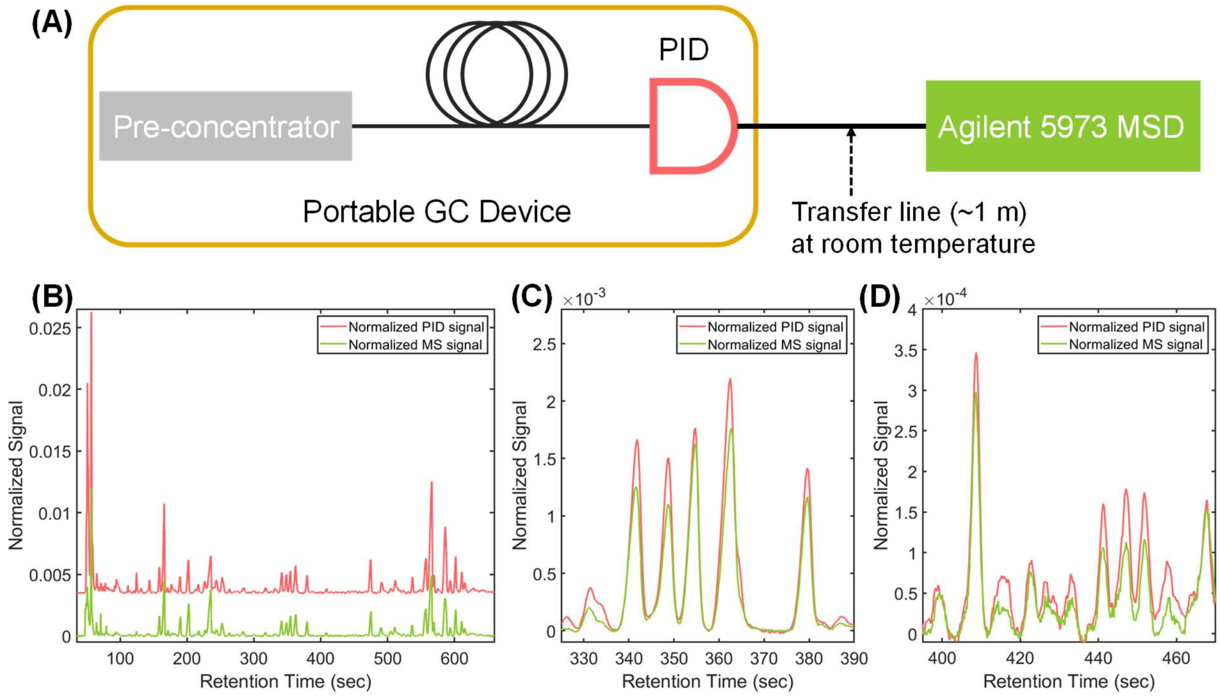

eFigure 3. (A) Setup that connects the outlet of our portable GC to an Agilent mass spectrometry (MS). (B) Chromatograms obtained concomitantly by the photoionization detector (PID) in our GC and MS for the same breath sample. For easy comparison, both chromatograms are normalized to the total area under the curve (from 0 s to 650 s). (C) and (D) Two zoom-in portions in (B).

© 2023 Sharma R et al. *JAMA Network Open.*

eFigure 4. Demographic Information of the Human Participants in the Study

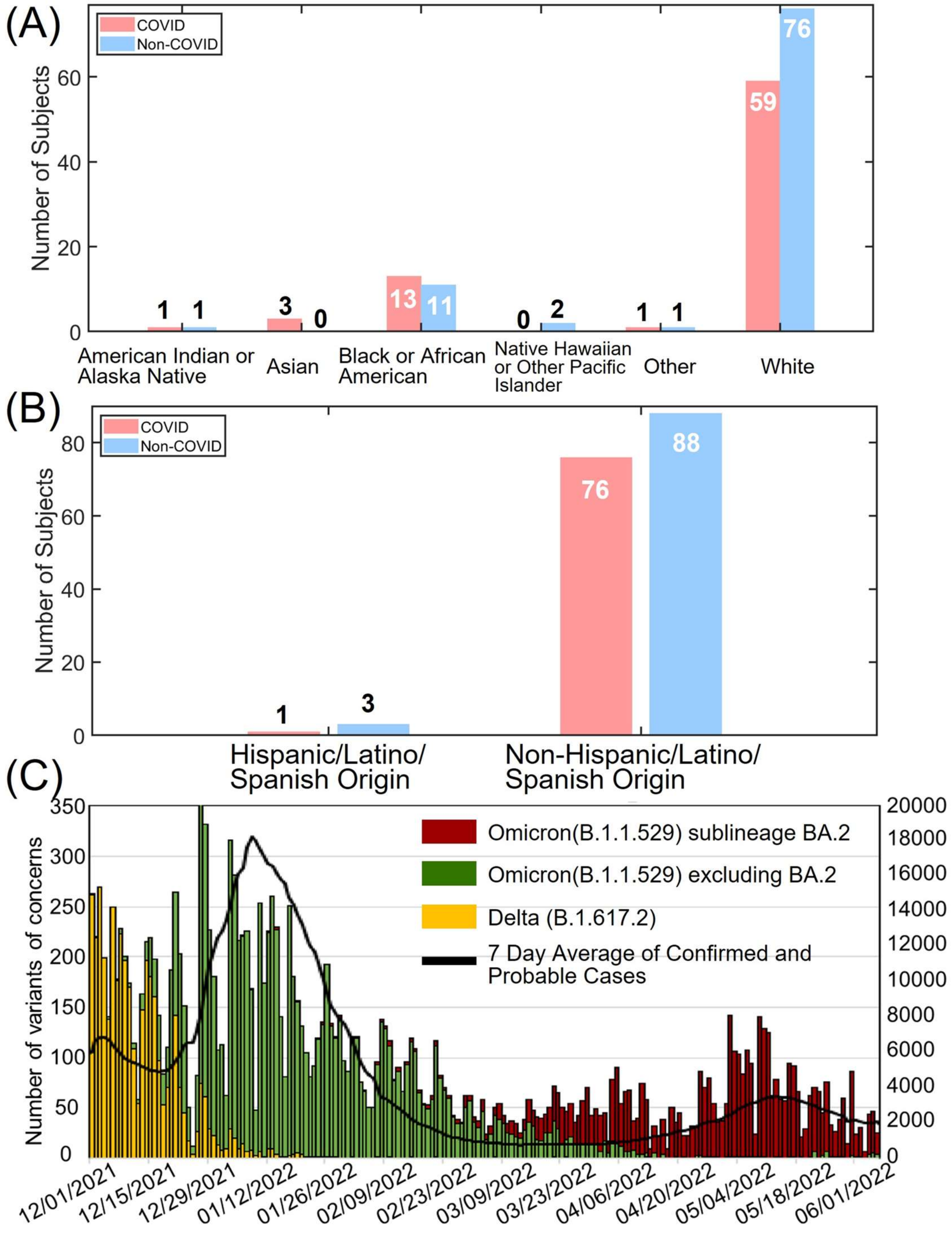

(C) shows the transition from Delta to Omicron in Michigan (adapted from Slide 8 in Ref. 4).

© 2023 Sharma R et al. *JAMA Network Open.*

eFigure 5. PCA Plot Using the 4 VOC Biomarkers in Figure 2 and Table 2 (in the Main Text) to Distinguish Between COVID-19 (2021) and Non–COVID-19

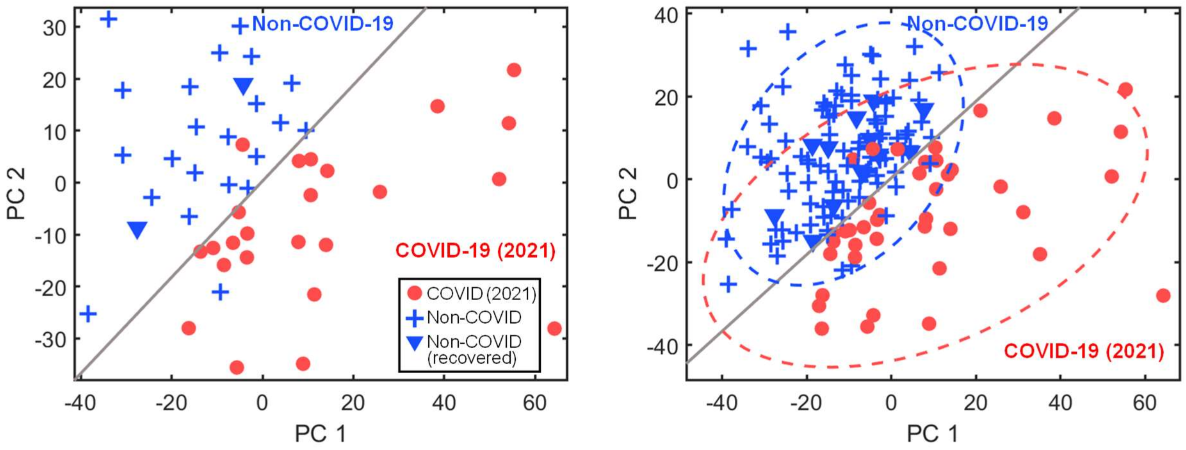

eFigure 5. PCA plot using the 4 VOC biomarkers in Figure 2 and Table 2 (in the main text) to distinguish between COVID-19 (2021) and non-COVID-19. (A) Training set. (B) Training set plus testing set. The corresponding statistics are given in Table 3 in the main text. COVID-19 (2021) patients (red circles) refer to those who were recruited prior to December 14, 2021, and were therefore assumed to be infected by Delta or earlier variants. Non-COVID-19 patients (blue crosses) refer to those who were recruited throughout the study (from April 26, 2021 to May 31, 2022). They were COVID-19 negative at the time when breath analysis was conducted and their COVID-19 infection history was unknown. Non-COVID-19 (recovered) patients (blue triangles) refer to those who were recruited throughout the study and had previously been COVID-19 positive (by RT-PCR), but recovered, *i.e.*, COVID-19 negative (by RT-PCR) at the time of breath analysis was conducted (and they are all correctly identified as COVID-19 negative by breath analysis). Each data point in the PCA plot represents one different breath sample. The breath sample was obtained and analyzed within 18 hours of the PCR test. The gray line marks the boundary of COVID-19 and non-COVID-19. The bottom right zone represents the COVID-19 region, whereas top left zone represents the non-COVID-19 region. The dashed curves are 95% confidence ellipses.

© 2023 Sharma R et al. *JAMA Network Open.*

eFigure 6. Violin Charts of the 4 Biomarkers Used in Figure 2 (in the Main Text) to Distinguish Between COVID-19 (2021) and Non–COVID-19

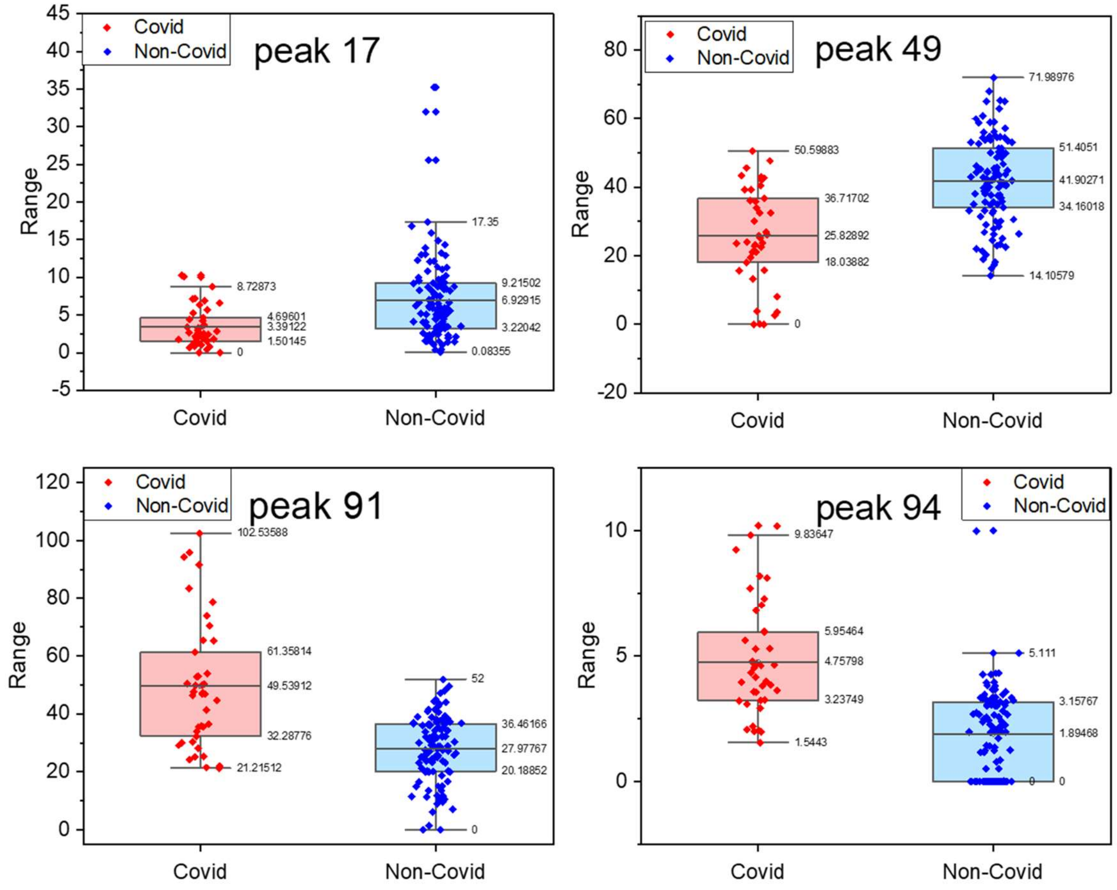

eFigure 6. Violin charts of the 4 biomarkers used in Figure 2 (in the main text) to distinguish between COVID-19 (2021) and non-COVID-19 (all COVID-19 negative recruited throughout the study). The PCA plot is shown in eFigure 5.

© 2023 Sharma R et al. *JAMA Network Open.*

eFigure 7. PCA Plot for Patient With COVID-19 (2022) When the 4 VOC Biomarkers in Figure 2 and Table 2 (in the Main Text) for COVID-19 (2021) Are Used

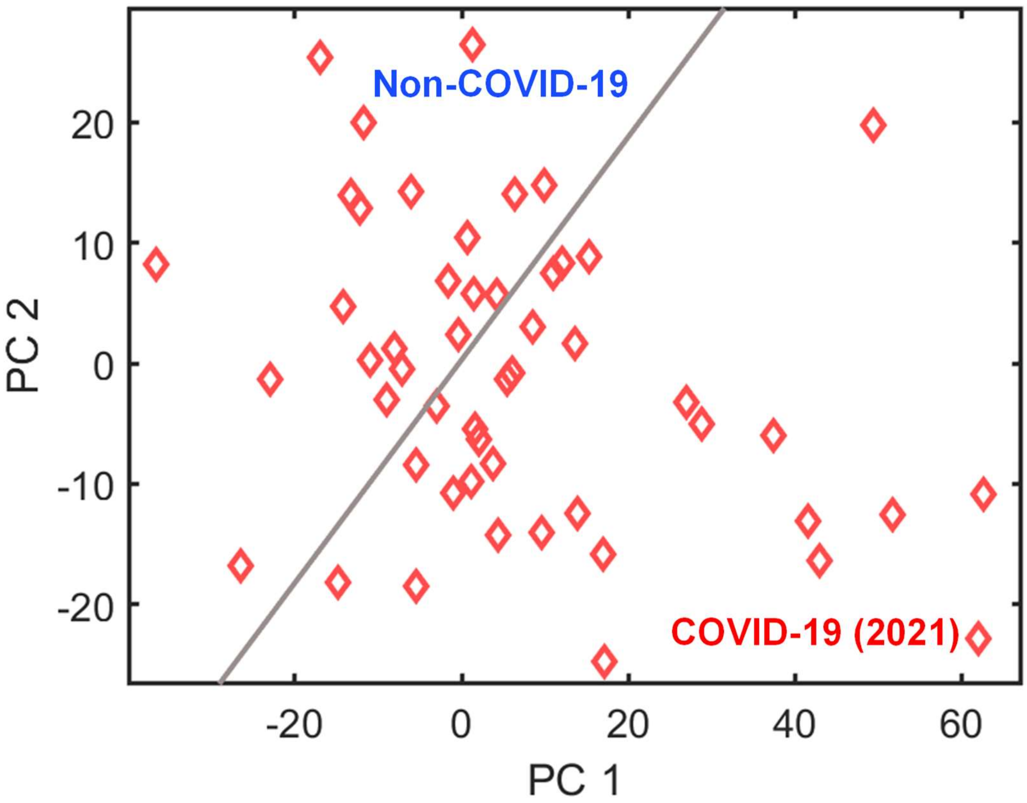

eFigure 7. PCA plot for COVID-19 (2022) patients when the 4 VOC biomarkers in Figure 2 and Table 2 (in the main text) for COVID-19 (2021) are used. A significantly lower specificity, sensitivity, and accuracy are obtained, which suggests that we cannot use the same set of biomarkers for Delta (and earlier variants) for Omicron. COVID-19 (2022) patients refer to those who were recruited after January 11, 2022 (till the end of the study – May 31, 2022) and were therefore assumed to be infected by Omicron. COVID-19 (2021) patients refer to those who were recruited prior to December 14, 2021and were therefore assumed to be infected by Delta and earlier variants.

© 2023 Sharma R et al. *JAMA Network Open.*

eFigure 8. Positions of the Biomarkers Used in eFigure 9 to Distinguish Between COVID-19 (2022) and Non–COVID-19 (Peak ID: 17, 67, 87, 97), in eFigure 10 to Distinguish Between COVID-19 (2021) and COVID19 (2022) (Peak ID: 27, 67, 69, 87, 94), and in eFigure 11 to Distinguish Between COVID19 (All Variants Occurring Between April 2021 and May 2022) and Non–COVID-19 (Peak ID: 12, 67, 84, 105)

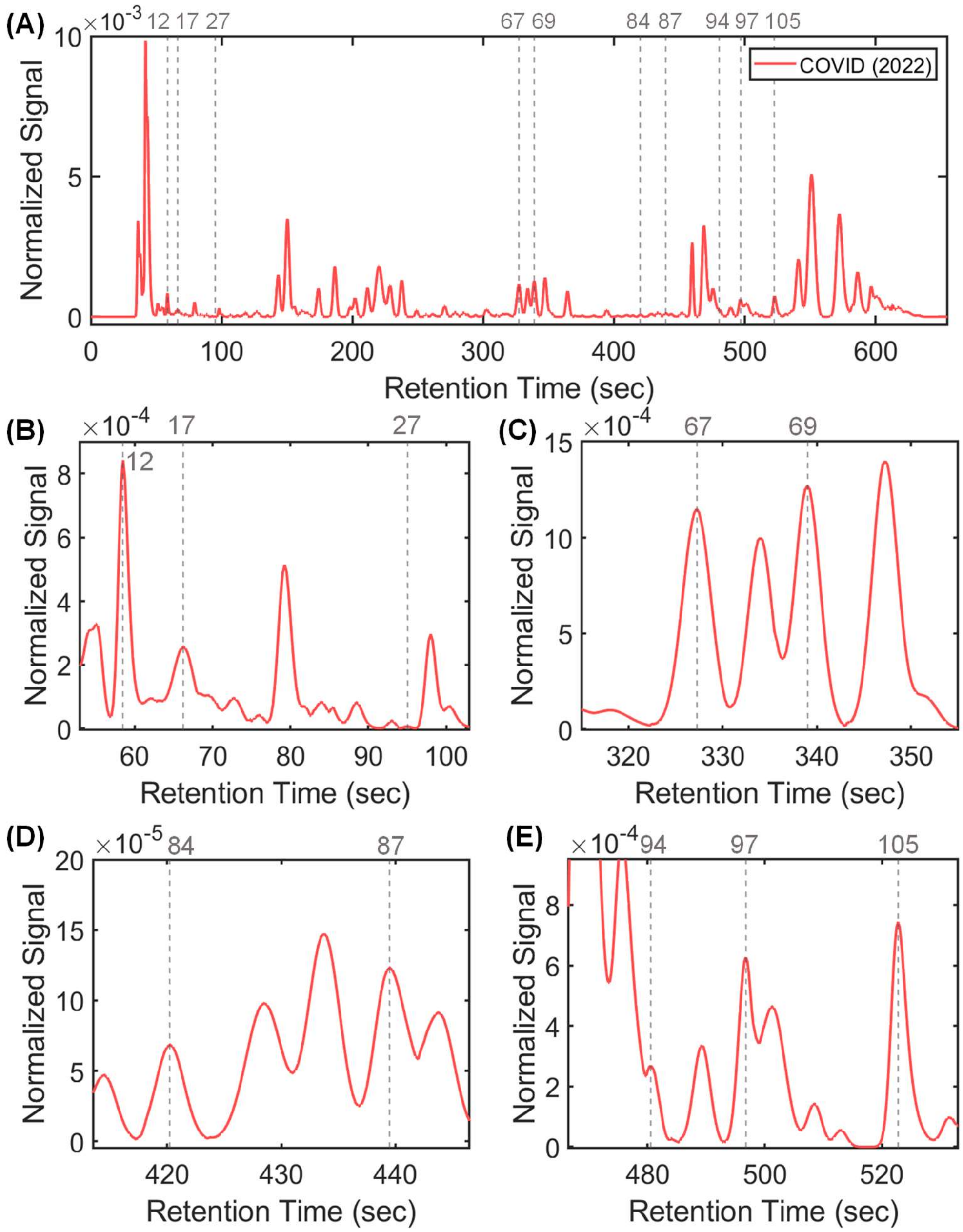

eFigure 8. Positions of the biomarkers used in eFigure 9 to distinguish between COVID-19 (2022) and non-COVID-19 (Peak ID: 17, 67, 87, 97), in eFigure 10 to distinguish between COVID-19 (2021) and COVID-19 (2022) (Peak ID: 27, 67, 69, 87, 94), and in eFigure 11 to distinguish between COVID-19 (all variants occurring between April 2021 and May 2022) and non-COVID-19 (Peak ID: 12, 67, 84, 105). The names of those VOCs are given in Table 2 in the main text.

© 2023 Sharma R et al. *JAMA Network Open.*

eFigure 9. PCA Plot Using 4 VOC Biomarkers in eFigure 8 and Table 2 (in the Main Text) to Distinguish Between COVID-19 (2022) and Non–COVID-19

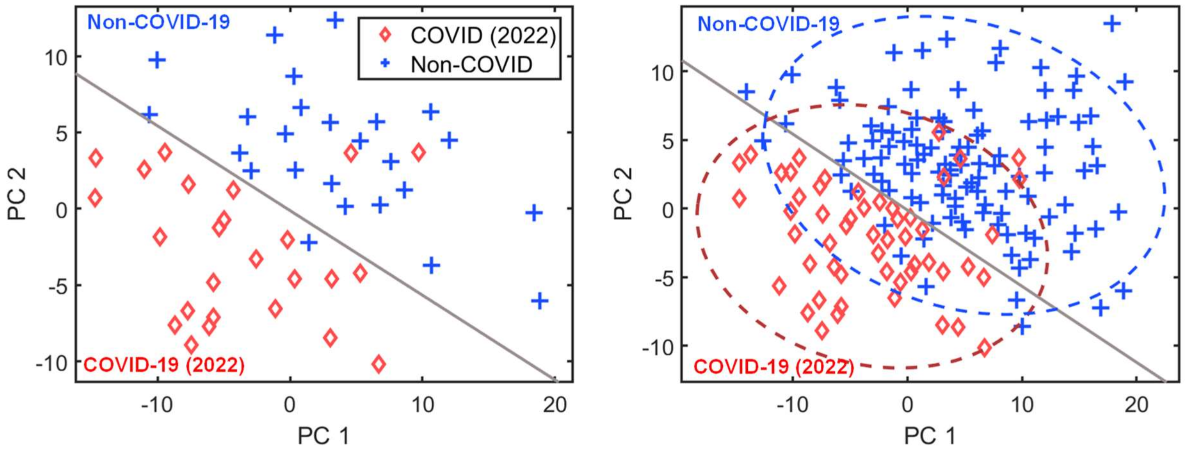

eFigure 9. PCA plot using 4 VOC biomarkers in eFigure 8 and Table 2 (in the main text) to distinguish between COVID-19 (2022) and non-COVID-19. (A) Training set. (B) Training set plus testing set. The corresponding statistics are given in Table 3 in the main text. COVID-19 (2022) patients (red diamonds) refer to those who were recruited after January 11, 2022 (until the end of the study – May 31, 2022) and were therefore assumed to be infected by Omicron. Non-COVID-19 patients (blue crosses) refer to those who were recruited throughout the study (from April 26, 2021 to May 31, 2022). They were COVID-19 negative at the time when breath analysis was conducted and their COVID-19 infection history was unknown, or recovered (*i.e.*, previously COVID-19 positive, but COVID-19 negative at the time when breath analysis was conducted). Each data point in the PCA plot represents one unique breath sample. The breath sample was obtained and analyzed within 18 hours of the RT-PCR testing. The gray line marks the boundary of COVID-19 and non-COVID-19. The bottom left zone represents the COVID-19 region, whereas the top right zone represents the non-COVID-19 region. The dashed curves are 95% confidence ellipses.

© 2023 Sharma R et al. *JAMA Network Open.*

eFigure 10. PCA Plot Using 5 VOC Biomarkers in eFigure 8 and Table 2 (in the Main Text) to Distinguish Between COVID-19 Omicron and Previous Variants

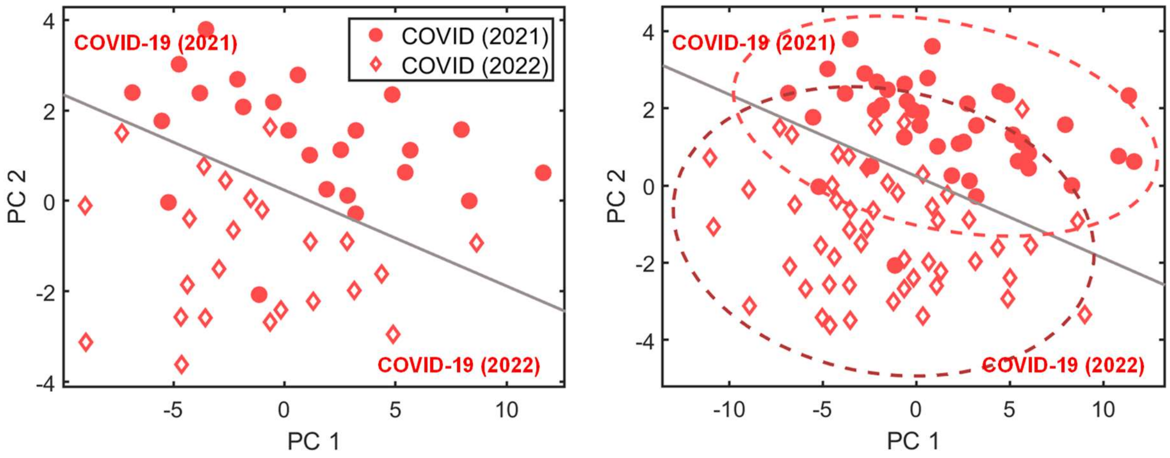

eFigure 10. PCA plot using 5 VOC biomarkers in eFigure 8 and Table 2 (in the main text) to distinguish between Omicron and the previous variants. (A) Training set. (B) Training set plus testing set. The corresponding statistics are given in Table 3 in the main text. Omicron patients, COVID-19 (2022), are denoted as red diamonds. They are the same as presented in eFigures 9. All patients of previous variants', COVID-19 (2021), are denoted as red circles. They are the same as presented in eFigures 5. Each data point in the PCA plot represents one different breath sample. The breath sample was obtained and analyzed within 18 hours of the PCR test. The gray line marks the boundary of Omicron (*i.e.*, COVID-19 (2022)) and previous variants (*i.e.*, COVID-19 (2021)). The bottom left zone represents the Omicron region, whereas the top right zone represents the previous variants region. The dashed curves are 95% confidence ellipses.

eFigure 11. PCA Plot Using 4 VOC Biomarkers in eFigure 8 and Table 2 (in the Main Text) to Distinguish Between COVID-19 (All Variants) and Non–COVID-19

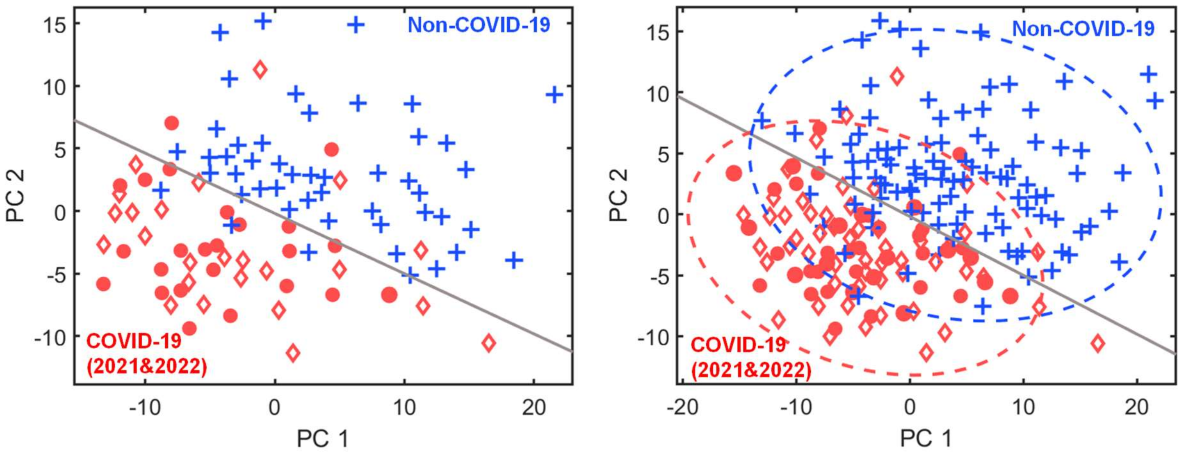

eFigure 11. PCA plot using 4 VOC biomarkers in eFigure 8 and Table 2 to distinguish between COVID-19 (all variants) and non-COVID-19. (A) Training set. (B) Training set plus testing set. The corresponding statistics are given in Table 3 in the main text. COVID-19 patients are denoted as red circles for COVID-19 (2021) and red diamonds for COVID-19 (2022), respectively. All non-COVID-19 patients are denoted as blue crosses. All the COVID-19 and non-COVID-19 (including the recovered) patients were recruited throughout the study from April 26, 2021 to May 31, 2022. Each data point in the PCA plot represents one different breath sample. The breath sample was obtained and analyzed within 18 hours of the PCR test. The gray line marks the boundary of COVID-19 and non-COVID-19. The bottom left zone represents the COVID-19 region, whereas the top right zone represents the non-COVID-19 region. The dashed curves are 95% confidence ellipses.

© 2023 Sharma R et al. *JAMA Network Open.*

eFigure 12. Trajectories on the PCA Plot for Various Patients Monitored for Multiple Days

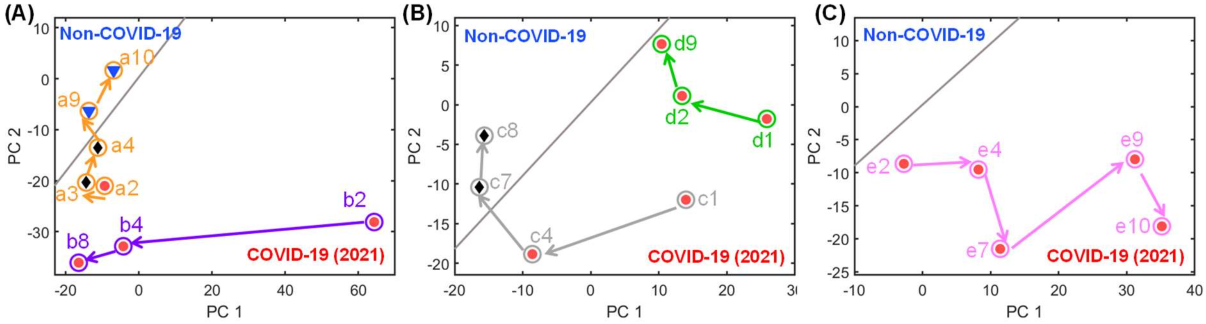

eFigure 12. Trajectories on the PCA plot for various patients monitored for multiple days. (A) and (B) are the recovery cases. The patients were initially COVID-19 positive, and later recovered and discharged from the hospital. Patient *b* on Day 2 (data point marked as *b*2) represents the most severe case among the entire patient pool, whose data point is the farthest in distance from the boundary (grey line). (C) A deterioration case. This patient (Patient *e*) was COVID-19 positive and his/her situation deteriorated over time. This patient died 21 days after our last GC measurement (Day 10). All the patients were recruited prior to December 14, 2021 and were therefore assumed to be infected by Delta or earlier variants. Each data point in the figure is denoted as "LowerCaseLetterNumber". For example, *b*2 refers to the data point of Patient *b*, whose breath collection/analysis was conducted on Day 2 after this patient was recruited into our study. Red circles and blue triangles denote COVID-19 positive and negative (recovered), respectively, which were confirmed by the PCR tests within 18 hours of breath collection/analysis. These data points also appear in the PCA plots in eFigure 5 and are tallied in Table 1 and statistics in Table 3 (in the main text). Black diamonds denote the breath data points that the corresponding PCR tests within 18 hours were not available. These data points are not plotted in eFigure 5, nor are they used/tallied in Table 1 or Table 3 (in the main text).

eFigure 13. Patients With Asymptomatic COVID-19 (2021) and Patients Who Were Infected by Other Viruses on the Same PCA Plot as in eFigure 5

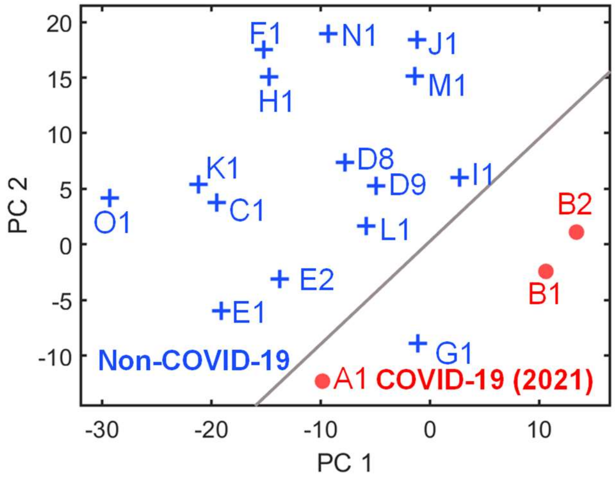

Patient	Patient ID	Virus type
A	121321A	COVID-19 (2021), asymptomatic
B	100521B	COVID-19 (2021), asymptomatic
C	072121A	HCoV-OC43
D	081021B	Rhinovirus
E	121421A	Rhinovirus
F	020822A	Human metapneumo virus
G	021022B	Parainfluenza virus 3
H	031022A	Rhinovirus
I	033022A	HCoV-OC43
J	033122B	Rhinovirus& Enterovirus
K	040122B	Parainfluenza virus 3
L	040522A	Rhinovirus&enterovirus, influenza A
M	041222B	Rhinovirus
N	042722A	HCoV-OC43
O	050922D	Rhinovirus

© 2023 Sharma R et al. *JAMA Network Open.*

eFigure 13. Patients With Asymptomatic COVID-19 (2021) and Patients Who Were Infected by Other Viruses on the Same PCA Plot as in eFigure 5. All COVID-19 positive patients (red circles) were recruited prior to December 14, 2021 and were therefore assumed to be infected by Delta or earlier variants. All non-COVID-19 patients (blue crosses) were recruited throughout the study (from April 26, 2021 to May 31, 2022). Each data point in the figure is denoted as "UpperCaseLetterNumber". For example, B2 refers to the data point of Patient B, whose breath collection/analysis was conducted on Day 2 after recruitment. The COVID-19 status of each data point was confirmed by the PCR test within 18 hours of breath collection/analysis. These data points have appeared in the PCA plots in eFigure 5 and tallied in Table 1 and statistics in Table 3 (in the main text). The types of viruses are listed in the table above.

© 2023 Sharma R et al. *JAMA Network Open.*

eFigure 14. Patients with Asymptomatic COVID-19 (2022) and Patients Who Were Infected by Other Viruses on the Same PCA Plot as eFigure 9

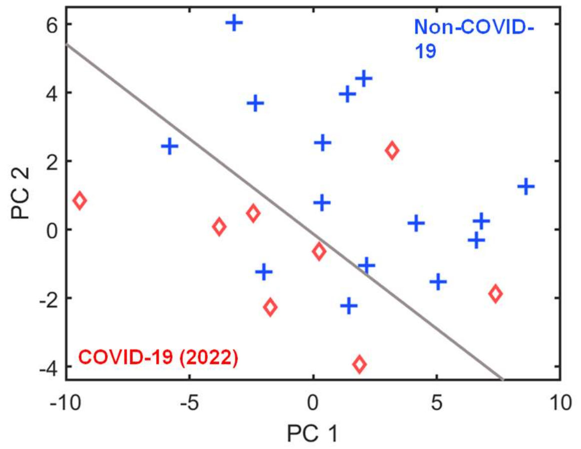

Patient	Patient ID	Virus type
P	020822B	COVID-19 (2022), asymptomatic
Q	022222A	COVID-19 (2022), asymptomatic
R	042522A	COVID-19 (2022), asymptomatic
S	042822D	COVID-19 (2022), asymptomatic
T	050922A	COVID-19 (2022), asymptomatic
U	051222D	COVID-19 (2022), asymptomatic
V	051622B	COVID-19 (2022), asymptomatic
W	051622D	COVID-19 (2022), asymptomatic
C	072121A	HCoV-OC43
D	081021B	Rhinovirus
E	121421A	Rhinovirus
F	020822A	Human metapneumo virus
G	021022B	Parainfluenza virus 3
H	031022A	Human Rhinovirus
I	033022A	HCoV-OC43
J	033122B	Rhinovirus& Enterovirus
K	040122B	Parainfluenza virus 3
L	040522A	Rhinovirus&enterovirus, influenza A
M	041222B	Rhinovirus
N	042722A	HCoV-OC43
O	050922D	Rhinovirus

© 2023 Sharma R et al. *JAMA Network Open.*

eFigure 14. Patients with Asymptomatic COVID-19 (2022) and Patients Who Were Infected by Other Viruses on the Same PCA Plot as eFigure 9. All COVID-19 positive patients (red diamonds) were recruited after January 11, 2022, and were therefore assumed to be infected by Omicron. All non-COVID-19 patients (blue crosses) were recruited throughout the study (from April 26, 2021 to May 31, 2022). Each data point in the figure is denoted as "UpperCaseLetterNumber", as in eFigure 13. The COVID-19 status of each data point was confirmed by the PCR test within 18 hours of breath collection/analysis. These data points have appeared in the PCA plots in eFigure 9 and tallied in Table 1 and statistics in Table 3 (in the main text). The types of viruses are listed in the table above.

© 2023 Sharma R et al. *JAMA Network Open.*

eFigure 15. Patients with Asymptomatic COVID-19 (Regardless of Variants) and Patients With Non–COVID-19 Who Were Infected by Other Viruses on the same PCA Plot as eFigure 11

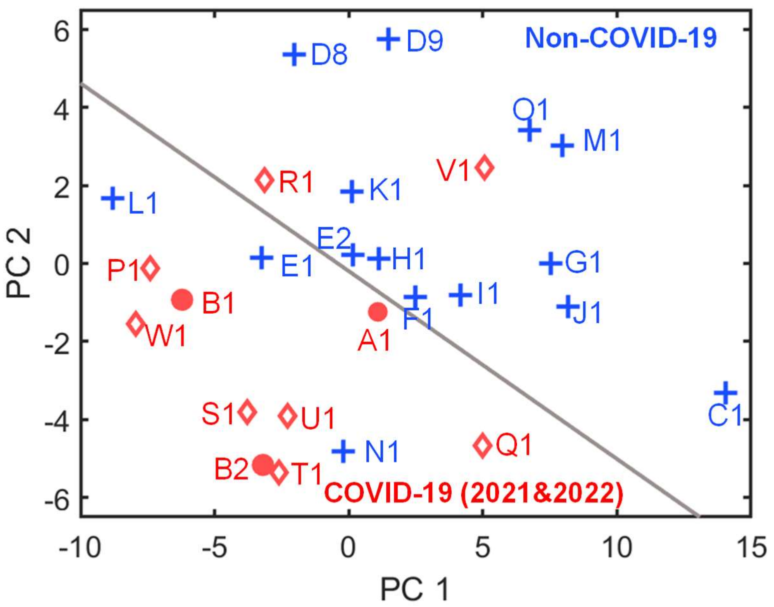

eFigure 15. Asymptomatic COVID-19 patients (regardless of variants, red dots and diamonds for COVID-19 (2021) and COVID-19 (2022), respectively) and non-COVID-19 patients who were infected by other viruses (blue crosses) on the same PCA plot as in eFigure 11. Each data point in the figure is denoted as "UpperCaseLetterNumber", as in eFigure 13. The COVID-19 status of each data point was confirmed by the PCR test within 18 hours of breath collection/analysis. These data points have appeared in the PCA plots in eFigure 11 and tallied in Table 1 and statistics in Table 3 (in the main text). The types of viruses are listed in the table in eFigure14.

© 2023 Sharma R et al. *JAMA Network Open.*

eTable. Four-Fold Cross-Validation

COVID-19 (2021) vs Non-COVID-19	Model 1	Model 2	Model 3	Model 4	Average
Specificity	96.4%	96.4%	92.9%	96.3%	95.2%
Sensitivity	90.0%	90.0%	90.0%	100.0%	93.3%
PPV	90.0%	90.0%	81.8%	91.7%	87.8%
NPV	96.4%	96.4%	96.3%	100.0%	97.6%
Total Accuracy	94.7%	94.7%	92.1%	97.4%	94.7%
**COVID-19 (2022) vs Non-COVID-19**	**Model 1**	**Model 2**	**Model 3**	**Model 4**	**Average**
Specificity	92.9%	92.9%	92.9%	88.9%	91.5%
Sensitivity	92.3%	92.3%	92.3%	78.6%	87.7%
PPV	85.7%	85.7%	85.7%	78.6%	83.3%
NPV	96.3%	96.3%	96.3%	88.9%	93.8%
Total Accuracy	92.7%	92.7%	92.7%	85.4%	90.2%
**COVID-19 (2021) vs COVID-19 (2022)**	**Model 1**	**Model 2**	**Model 3**	**Model 4**	**Average**
Specificity	92.3%	84.6%	100.0%	92.9%	92.5%
Sensitivity	90.0%	100.0%	90.0%	90.9%	93.6%
PPV	90.0%	83.3%	100.0%	90.9%	91.4%
NPV	92.3%	100.0%	92.9%	92.9%	95.2%
Total Accuracy	91.3%	91.3%	95.7%	92.0%	93.0%
**COVID-19 (2021&2022) vs Non-COVID-19**	**Model 1**	**Model 2**	**Model 3**	**Model 4**	**Average**
Specificity	89.3%	89.3%	96.4%	88.9%	91.5%
Sensitivity	91.7%	87.5%	87.5%	86.4%	87.1%
PPV	88.0%	87.5%	95.5%	86.4%	89.8%
NPV	92.6%	89.3%	90.0%	88.9%	89.4%
Total Accuracy	90.4%	88.5%	92.3%	87.8%	89.5%

eTable. To test the model robustness, 4-fold cross validation with all 4 categories, including (1) COVID-19 (2021) vs Non-COVID-19, (2) COVID-19 (2022) vs Non-COVID-19, (3) COVID-19 (2021) vs COVID-19 (2022), and (4) COVID-19 (2021, 2022) vs Non-COVID-19 were conducted. The number of samples in (1) was 152, including 41 COVID-19 (2021) samples and 111 Non-COVID-19 samples. The number of samples in (2) was 164, including 53 COVID-19 (2022) samples and 111 Non-COVID-19 samples. The number of samples in (3) was 94, including 41 COVID-19 (2021) samples and 53 COVID-19 (2022) samples. The number of samples in (4) was 205, including 41 COVID-19 (2021) samples, 53 COVID-19 (2022) samples, and 111 Non-COVID-19 samples. The samples in each category were randomly divided into 4 subsets of equal or similar size to generate the corresponding model, in which 75% were used as the training set and the remaining 25% samples were used as the testing set. Note that the data sets used in this 4-fold cross validation might contain multiple breath samples from the same patient measured over multiple days, as discussed previously in the main text, whereas in the training sets presented in Table 3 in the main text, only a single breath sample was selected from each patient.

eReferences

1. Sharma R, Zang W, Zhou M, et al. Real Time Breath Analysis Using Portable Gas Chromatography for Adult Asthma Phenotypes. *Metabolites.* 2021;11:265.
2. Zhou M, Sharma R, Zhu H, et al. Rapid Breath Analysis for Acute Respiratory Distress Syndrome Diagnostics Using a Portable Two-Dimensional Gas Chromatography Device. *Anal. Bioanal. Chem.* 2019;411:6435-6447.
3. Sharma R, Zhou M, Tiba MH, et al. Breath Analysis for Detection and Trajectory Monitoring of Acute Respiratory Distress Syndrome in Swine. *Eur. Respir. J. Open Res.* 2022;8:00154-02021.
4. *www.michigan.gov/coronavirus/-/media/Project/Websites/coronavirus/Michigan-Data/Data-and-Modeling-Updates/20220621-Data-and-modeling-update_vFINAL.pdf?rev=e6f902ee8e8d4a969336c1bc1a049008&hash=5FF667FAEE5F5A175CA304F1FC125CB7*.

© 2023 Sharma R et al. *JAMA Network Open.*
