# Supplementary material for: Portable Breath-Based Volatile Organic Compound Monitoring for the Detection of COVID-19 During the Circulation of the SARS-CoV-2 Delta Variant and the Transition to the SARS-CoV-2 Omicron Variant
Source: JAMA Netw Open. 2023 Feb 28;6(2):e230982. doi: 10.1001/jamanetworkopen.2023.0982 (PMC9975913; doi:10.1001/jamanetworkopen.2023.0982)
Supplement: Supplement 2. — Data Sharing Statement [file jamanetwopen-e230982-s002.pdf]

Data Sharing Statement

Sharma. Portable Breath-Based Volatile Organic Compound Monitoring for the Detection of COVID-19 During the Circulation of the SARS-CoV-2 Delta Variant and the Transition to the SARS-CoV-2 Omicron Variant. *JAMA Netw Open*. Published February 28, 2023.
doi:10.1001/jamanetworkopen.2023.0982

Data
Data available: Yes
Data types: Data dictionary
How to access data: https://radx-hub.nih.gov/home
When available: With publication

Supporting Documents
Document types: None

Additional Information
Who can access the data: Anyone requesting
Types of analyses: Any purpose
Mechanisms of data availability: Through NIH data hub without investigator support
Any additional restrictions: N/A
